# Supplementary figures and images for: A method to estimate the cellular composition of the mouse brain from heterogeneous datasets
Source: PLoS Comput Biol. 2022 Dec 21;18(12):e1010739. doi: 10.1371/journal.pcbi.1010739 (PMC9838873; doi:10.1371/journal.pcbi.1010739)

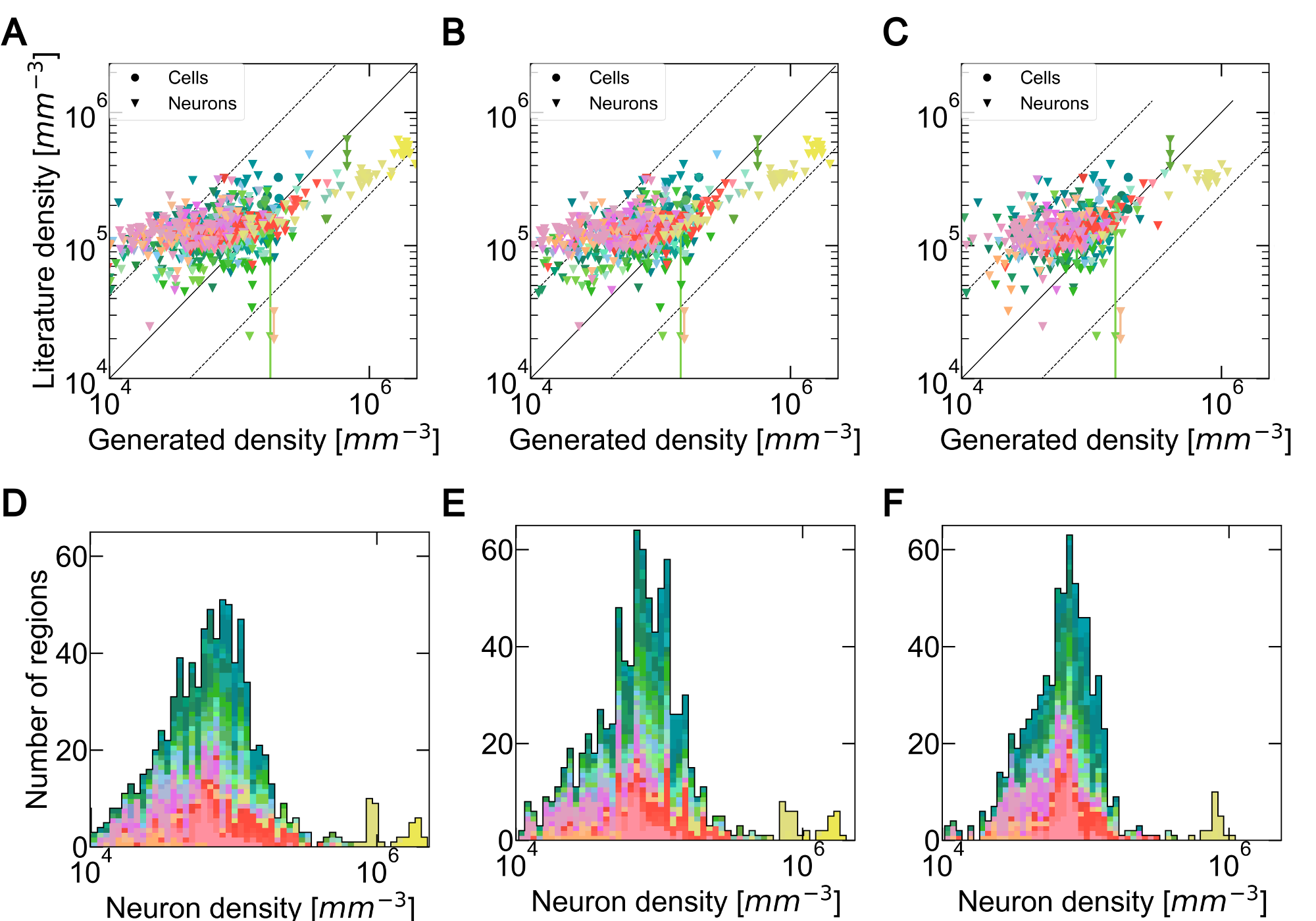

Supplement: S1 Fig — (A), (B), (C) Distribution of cell and neuron density values reported in literature against generated densities for similar regions, using the 3 pairs of annotations and Nissl volumes (from left to right: CCFbbp, CCFv2 from the AIBS, CCFv3 from the AIBS). When multiple literature sources are available for the exact same region, they are both shown as a data point and are linked together. The color encodes the brain regions according to the AV, while the shapes of the points encode for cell types. The middle line delimits equal quantities, while the dashed line shows the average deviation of 2.7-fold between literature values reporting on the same region. Some subregions of the brain are not represented in CCFv3 which explains the different numbers of points. (D), (E), (F) Histogram of the brain regions in terms of neuron density values for the 3 pairs of AV and Nissl volumes (from left to right: CCFbbp, CCFv2 from the AIBS, CCFv3 from the AIBS). Each region is represented with a single-color patch of the same size. (TIF) [file pcbi.1010739.s001.tif]

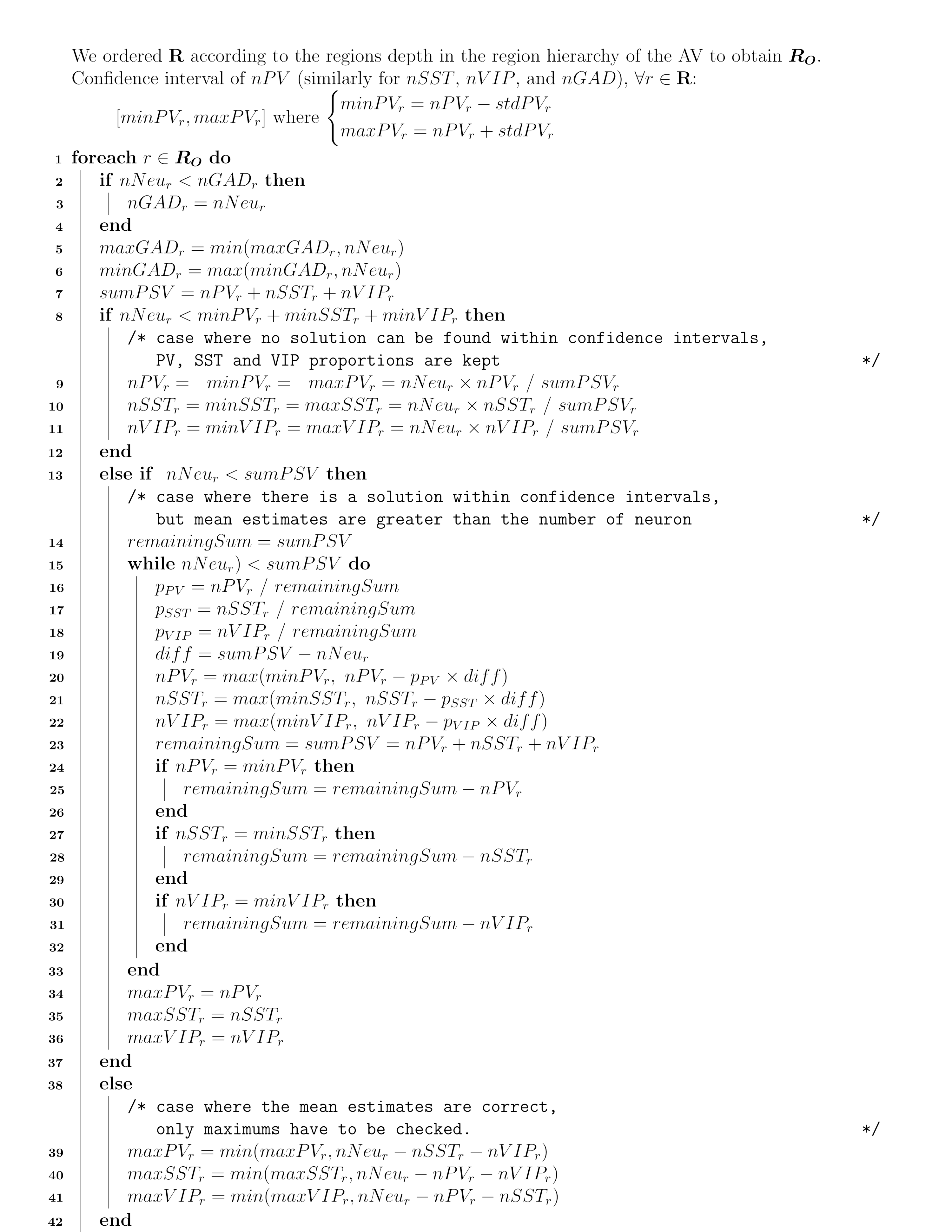

Supplement: S2 Fig — The estimated counts of GAD67+ neurons and the sum of PV+, SST+, VIP+ neurons counts are limited by the previously-computed neuron counts (step 2 of the BBCAv2 pipeline—see Fig 1) to ensure that assumptions 2 and 3 are fulfilled (see Section 2.4). Recall that R is the set of brain regions, ∀ r ∈ R inversely ordered according to their depth in the region hierarchy of the AV (Ro), the algorithm checks if the conditions nNeur ≥ nGADr and nNeur ≥ nPVr + nSSTr + nVIPr are satisfied. If not, it finds a solution which tries to match the following properties, ordered by priority: (1): Remain in range of the standard deviation of each value (confidence intervals), (2): Maintain the proportion of PV, SST and VIP within the region. If a solution exists within the confidence intervals, then the number of extra neurons (diff variable at line 19) is subtracted proportionally to the ratio of each neuron type (computed at lines 16–18). Then, if one of the neuron type estimates reaches the minimum of its confidence interval, then it is no more reduced (lines 20–22) and the remaining extra neurons are subtracted from the other neuron type estimates (lines 25, 28, 31). (TIF) [file pcbi.1010739.s002.tif]

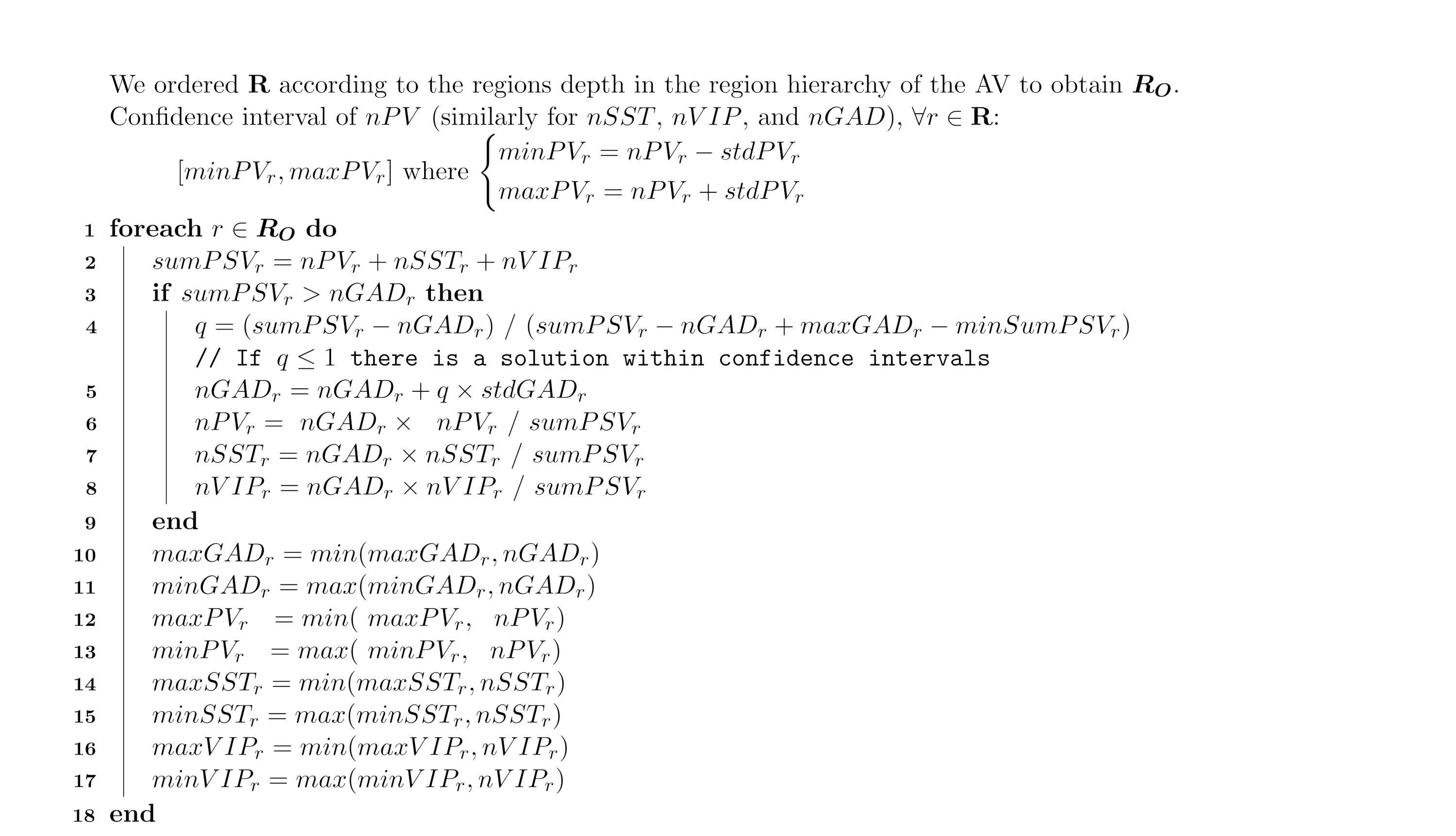

Supplement: S3 Fig — This algorithm corrects the estimated densities of PV+, SST+, VIP+ and GAD67+ neurons in the model so that assumption 4 of Section 2.4 is fulfilled. R is the set of brain regions, ∀ r ∈ R inversely ordered according to their depth in the region hierarchy of the AV (RO), the algorithm checks if the condition nGADr ≥ nPVr + nSSTr + nVIPr is satisfied. If not, it finds a solution which tries to match the following properties, ordered by priority: (1): Remain in range of the standard deviation of each value (confidence intervals), (2): Maintain the proportion (ratios) of PV, SST and VIP. When a set of value exists so that the property (1) is fulfilled, there is a correction factor q ∈ [0,1] which corresponds to the fraction of standard deviation needed to guarantee that the sum of the inhibitory subtypes remains under the estimated count of inhibitory neurons and that each value remains within its confidence interval. (TIF) [file pcbi.1010739.s003.tif]

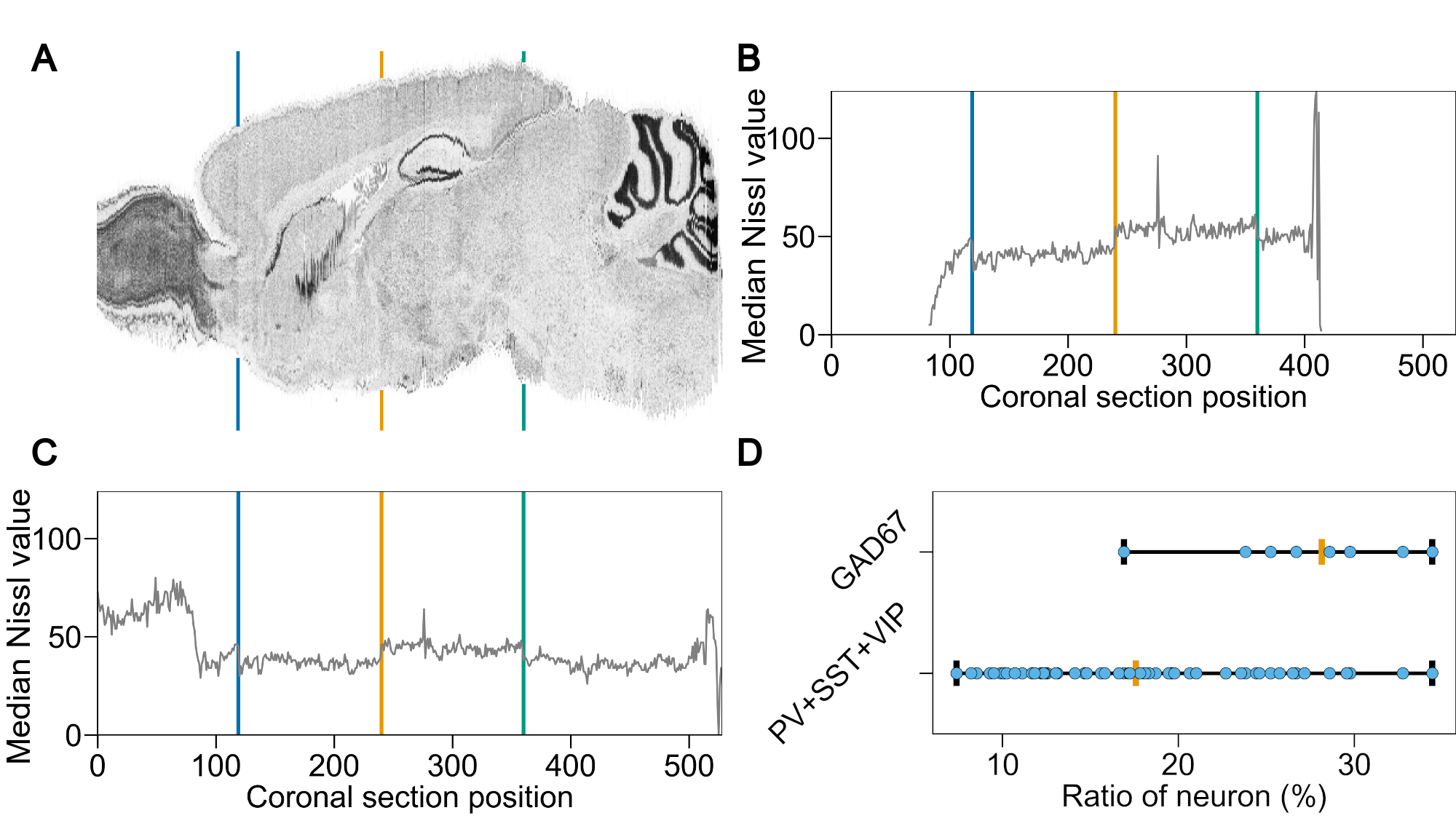

Supplement: S4 Fig — (A) Coronal view of the Nissl reference atlas used for BBCAv2, showing cells of the mouse brain. Regions with high cell density appear in dark grey. The blue, orange and green lines behind the sagittal slice highlight the rapid changes of Nissl expression coming from the original Nissl experiment from Dong [18]. (B) (C) Evolution of the median expression level along the sagittal axis, in the Nissl volume realigned in Erö et al. [17], for the whole brain (B) and the isocortex (C). The rapid changes of Nissl expression that were detected in (A) are also visible in (B) and (C). (D) isocortex ratio of inhibitory neurons according to literature. The distributions show to ratios literature cell type counts in isocortex divided by the counts of neurons of the BBCAv2 in their corresponding region. The bottom and top distributions correspond to the proportion of respectively the sum of the reported values of PV, SST and VIP counts, and the reported values of GAD67. The mean value of each distribution is shown in orange. The minimum and maximum values are indicated by the whiskers. (TIF) [file pcbi.1010739.s004.tif]

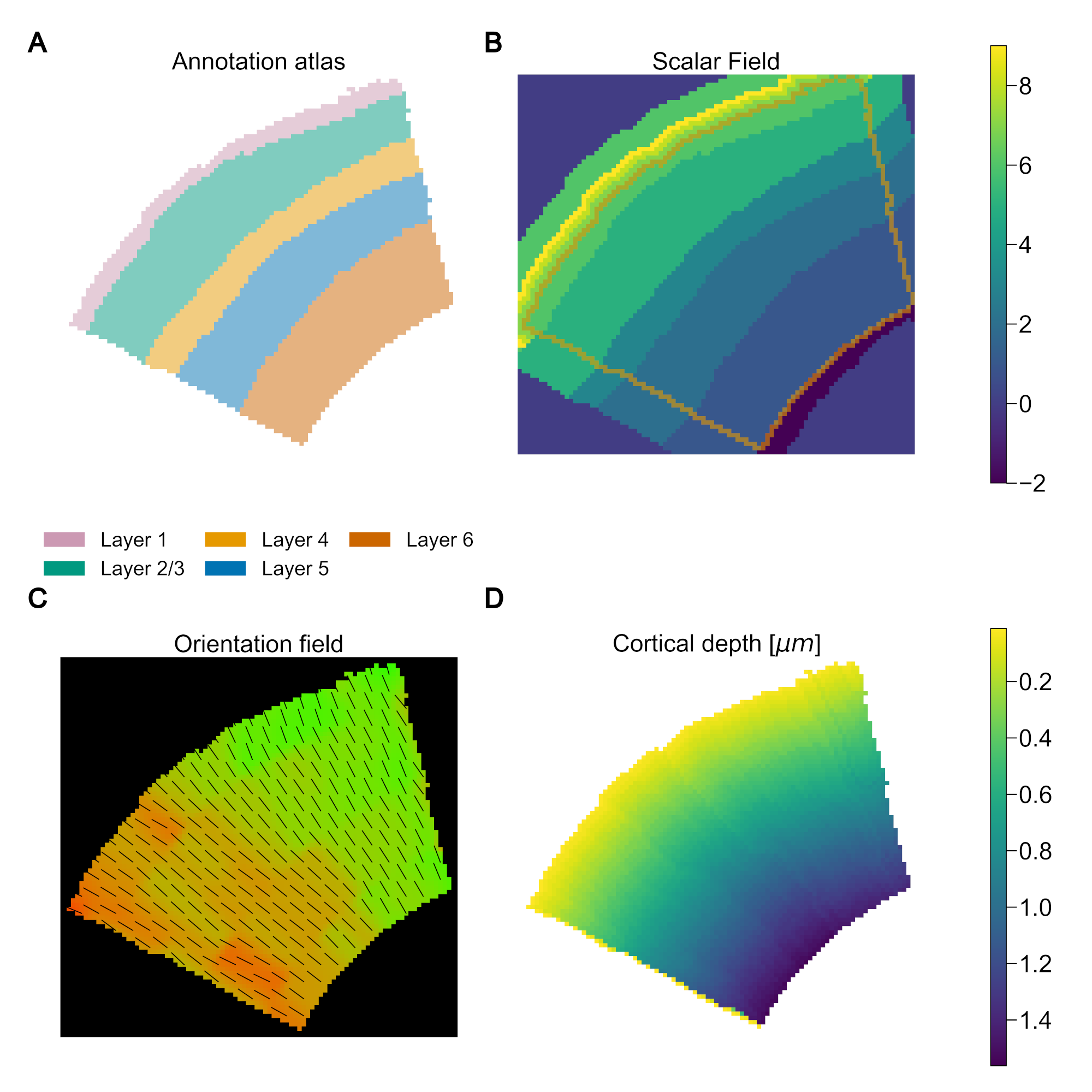

Supplement: S5 Fig — (A) Coronal slice of the AV showing the barrel cortex and its different sublayers. (B) Coronal slice of the scalar field of the barrel cortex. A weight is assigned to every voxel of the AV. These weights follow the order of crossing the isocortex by its fibers from the corpus callosum to layer 1. The borders of the barrel field are highlighted in orange. The weight assigned to the surrounding voxels of the region corresponds to their closest layer’s. The weight of the voxels outside the region beyond layer 1 increases as moving away from the barrel cortex. (C) Coronal slice of the orientation field of the barrel cortex. To each voxel of the AV, a 3D direction normalized vector is computed corresponding to the main axis of the axons in the region. Colors represent the orientation vectors norm on their respective plane, black lines their projected axis. (D) Coronal slice of the depth according to pia in the barrel cortex expressed in micrometers. (TIF) [file pcbi.1010739.s005.tif]

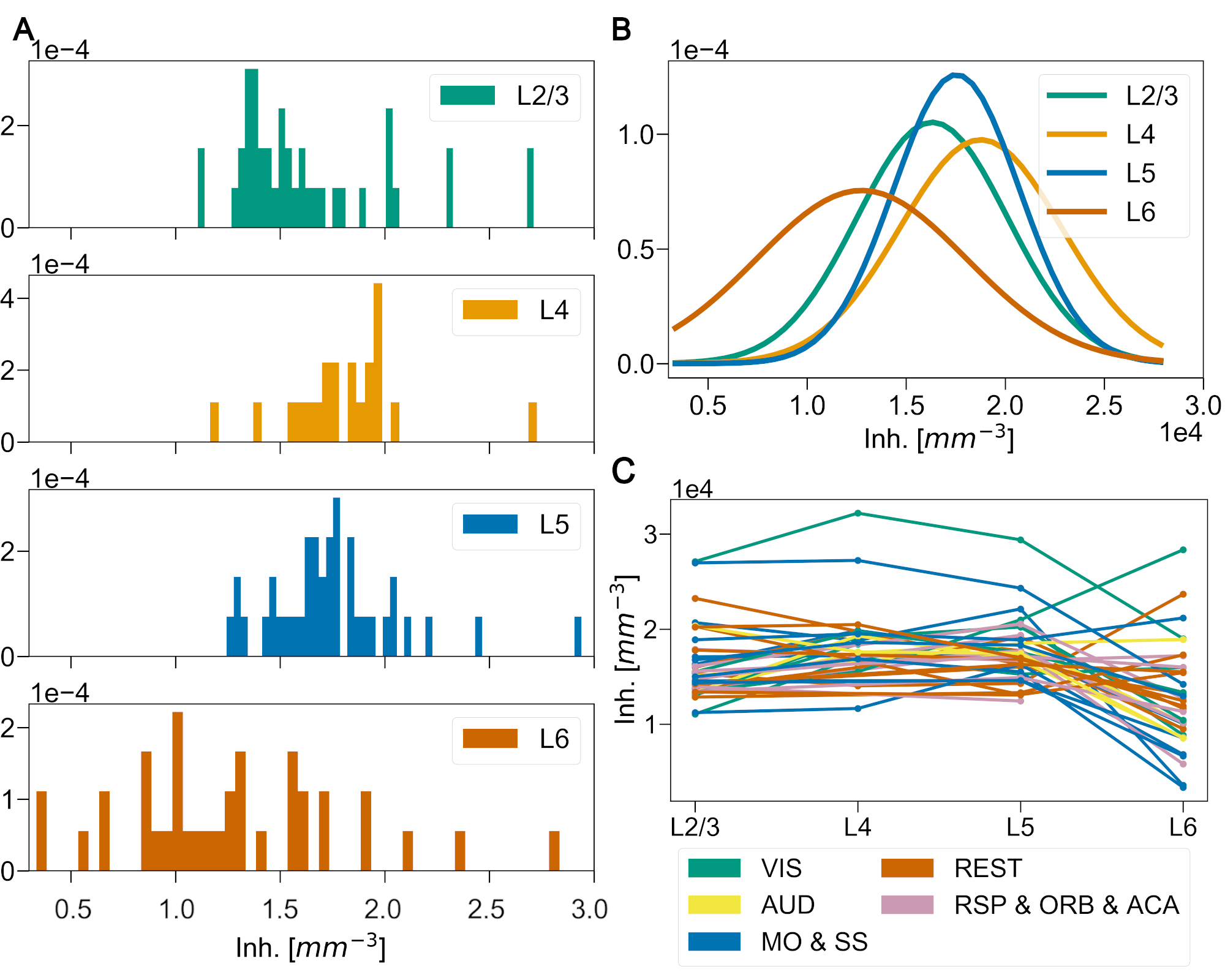

Supplement: S6 Fig — (A) Normalized distribution of cortical inhibitory neuron densities across the isocortex subregions grouped by layers. For each layer, the fitted normal distribution associated is displayed as a line on top of it. The normal distributions of L2/3 (mean 1.6x10^4^, std. 3.8 x10^3^), L4 (mean 1.9x10^4^, std. 4.1 x10^3^) and L5 (mean 1.8x10^4^, std. 3.1 x10^3^) are overlapping significantly. (B) Distribution of inhibitory neuron densities according to cortical layers for each subregion of the isocortex. For most of the cortical subregions, the density is constant from L2 to L5 and drops for L6. (TIF) [file pcbi.1010739.s006.tif]

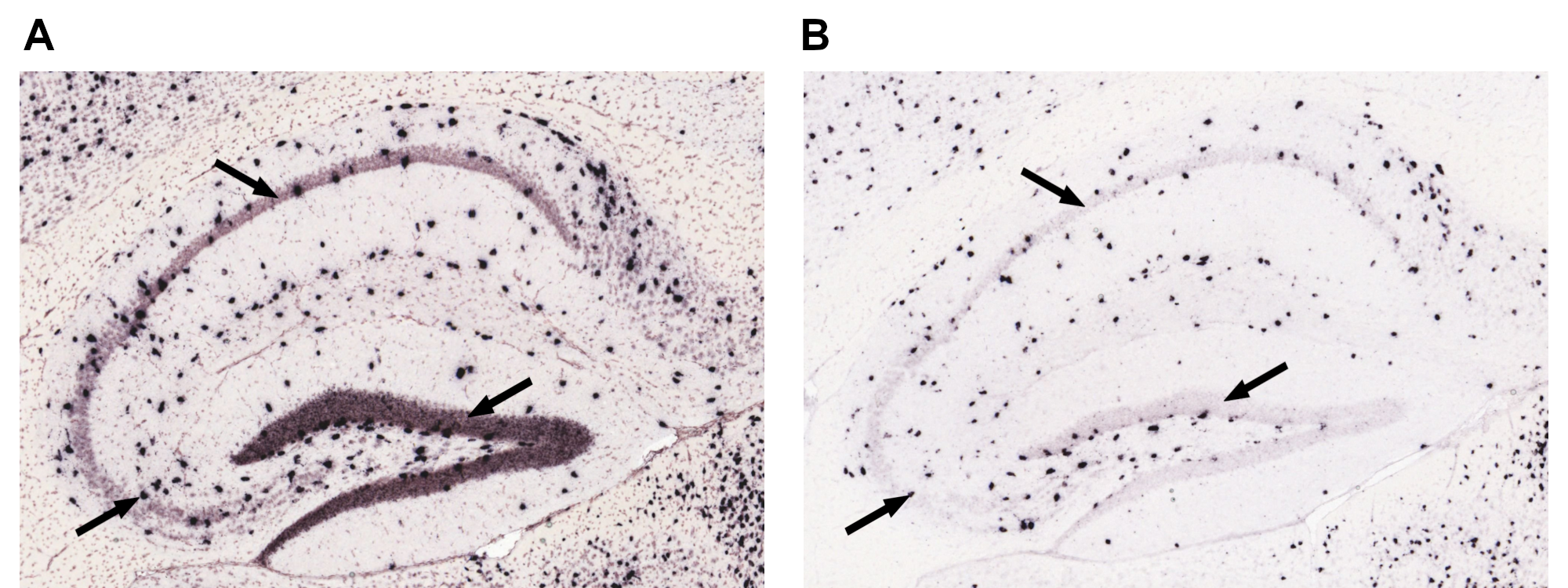

Supplement: S7 Fig — GAD67 (A) and GAD65 (B) ISH sagittal slices (images are respectively the 14th slice of experiment #75457536 and the 11th slice of experiment #79903740) of the mouse brain from the AIBS website, showing the dentate gyrus (region resembling a greater-than symbol). Somas reacting to the marker stand out from the background. The more a cell is reacting to the marker the darker it will be shown in the image. Two different populations of cells reacting to the markers can be seen in the images. First, a population of cells with large somas, and strongly reacting to both the GAD67 and GAD65 markers. Second, a dense population of cells with small somas, reacting to the GAD67 marker but not to GAD65. (TIF) [file pcbi.1010739.s007.tif]

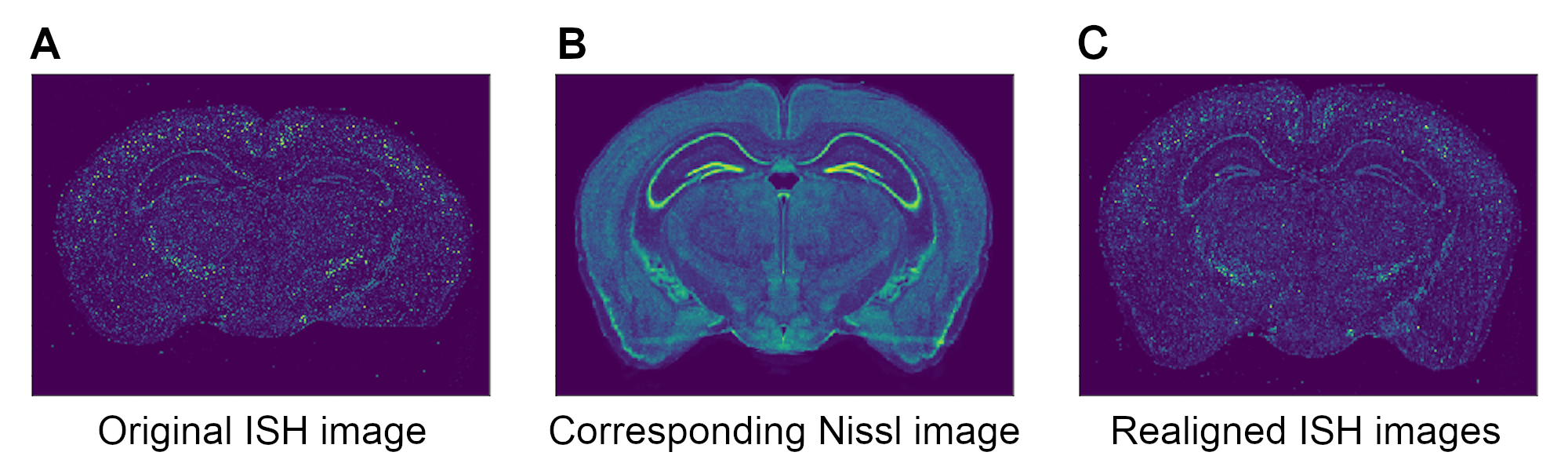

Supplement: S8 Fig — This figure shows coronal slices of the mouse brain from AIBS experiments of Nissl and PV (experiment id #868). ISH images from the AIBS (A) are realigned to their corresponding slice in the Nissl volume (B) using the Krepl et al.’s algorithm [56]. The registration is performed on the raw images from the AIBS as they provide more landmarks and then applied to the filtered images (C). (TIF) [file pcbi.1010739.s008.tif]

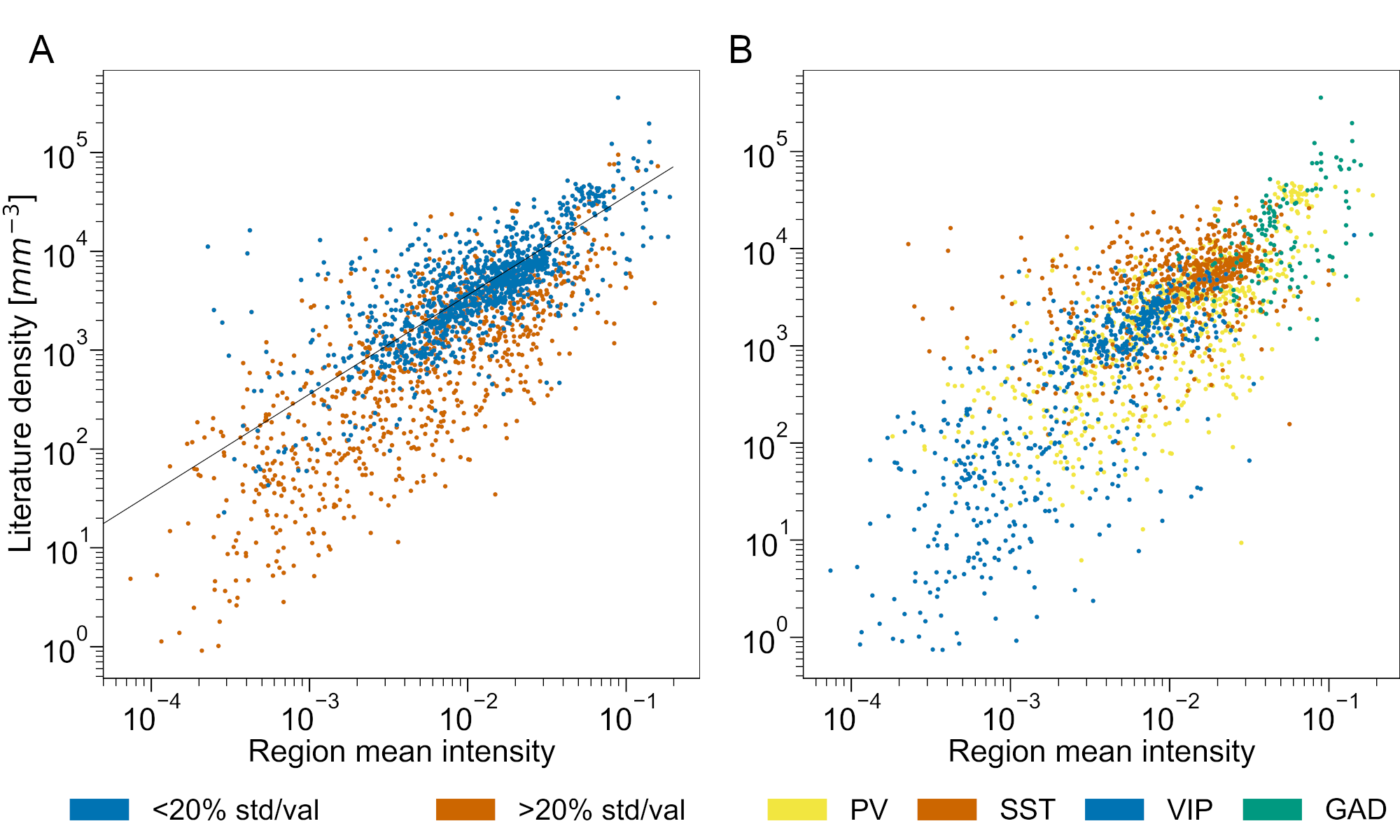

Supplement: S9 Fig — Scatter plots of the PV+, SST+, VIP+ and GAD+ densities reported in literature (y-axis) according to the region mean intensity (x-axis). Each point represents a single literature density value. (A) The scatter plot is color-coded according to the different levels of confidence from literature data (ratio of standard deviation over mean value). The linear fit is represented with a black line. (B) Same scatter plot as (A) but the points are here color-coded according to the genetic marker expressed by the neuron population. (TIF) [file pcbi.1010739.s009.tif]
