# Supplementary material for: A method to estimate the cellular composition of the mouse brain from heterogeneous datasets
Source: PLoS Comput Biol. 2022 Dec 21;18(12):e1010739. doi: 10.1371/journal.pcbi.1010739 (PMC9838873; doi:10.1371/journal.pcbi.1010739)
Supplement: S1 Document — This document contains the supplementary methods compute cell orientations and the literature review. It also contains the table of abbreviations used in this paper (Table A). (DOCX) [file pcbi.1010739.s012.docx]

**Supplementary materials**

[Supplementary methods 1](#_Toc119362330)

[**Orientations field and depth processing** 1](#_Toc119362331)

[**Review of literature on densities of inhibitory neuron in the mouse brain** 4](#_Toc119362332)

[**Finding an initial solution for the optimization** 9](#_Toc119362333)

[Supplementary Tables 11](#_Toc119362334)

[**Table A. Nonstandard abbreviations** 13](#_Toc119362335)

[References 14](#_Toc119362336)

# Supplementary methods

## **Orientations field and depth processing**

Processing the distance towards locations in the BBCA is crucial for *in-silico* modeling. For instance, assigning morphologies to cells requires knowing the space available for them [1]. Similarly, several literature reports depth-based density [2] or connectivity rules [3], which makes it more difficult to integrate them into brain models. In this paper, we validated our density profiles of the barrel field against the results from Meyer [4] which requires to compute the distance of each voxel of the AV towards the pia, guided by the orientation field of the main fibers axis in the isocortex (see S5D Fig).

To obtain this orientation field, we created a semi-automated method to generate coordinate systems using only a user-defined list of brain regions as reference. The goal was to compute a vector field pointing towards a general direction, based on a source region, a target region. Within the region of interest, direction vectors are obtained as the normalized gradient of a scalar field. Which in turn is obtained by assigning to every voxel of the brain a user-defined weight representing its distance from the source region (see S5A and S5B Fig). For each subregion of interest, a single weight is assigned to every voxel of that subregion and a default value is given to the rest of the brain. For the isocortex, the following weights were assigned to the voxel of the scalar field:

- -2 for the lateral forebrain bundle system (corpus callosum)
- 1 for the layer 6
- 2 for the layer 5
- 3 for the layer 4
- 4 for the layer 3
- 5 for the layer 2 (and 2 / 3)
- 6 for the layer 1
- 0 for the other brain regions

To avoid boundary effects from the outside at the borders of a region, we extended the scalar field of the region to its surroundings voxels (see S5B Fig). These surrounding voxels are detected by a shading algorithm which looks for voxels close to annotation borders (i.e., where a change of annotation is occurring). We applied this shading algorithm to each layer of the isocortex, setting their surrounding voxels to the same weight in the scalar field.

An additional scalar shading is computed based on the distance to a subregion of interest identified as a target for fibers. This shading was created to attract the gradient of the voxels in the target region towards the outside. For the isocortex, voxels closed to the L1 and outside of the brain were assigned to 6 plus their distance to L1.

A Gaussian filter is then applied to the initialized scalar field and the gradient of the normalized blurred scalar field is eventually returned. The direction vectors are given by this gradient (see S5C Fig). This process was applied to all cortical areas by defining the white matter as the source region, and the outside of the brain as the target.

The orientation field algorithm can also be applied to the subregions of the cornu ammonis (CA), where the source and target regions are defined respectively as the stratum radiatum and stratum oriens. Similarly, it could be used in the Cerebellar Cortex defining the source region as the arbor vitae and the target region as the molecular layer.

The process of finding depth and boundaries for a voxel follows a straightforward procedure:

- Starting from the position of the voxel, add the unit vector corresponding to the orientation field axis (direction of pia) and check the layer at the new position.
- If the layer is different after the step, a boundary has been crossed and we record the distance traveled to get to it.
- Repeat this process until the isocortex has been exited.

The same can be done by subtracting the unit vector from the position (direction of white matter) to get the locations of the boundaries of deeper layers. Finally, a mean layer boundary distance to the pia can be extracted from the distance of its voxels.

## **Review of literature on densities of inhibitory neuron in the mouse brain**

We performed a systematic review of the literature for inhibitory neurons in the mouse brain. To do so, we mostly leveraged the google scholar tool to find articles. Our searches involved combinations of the following key words (and their respective common abbreviations, e.g.: GAD67 for GABAergic): mouse, neurons, cells, inhibitory, GABAergic, parvalbumin, somatostatin, vasoactive intestinal peptide, interneurons, quantitative analysis, counts, densities, etc. We additionally searched more specifically for specific regions of the brain to confirm findings or try to improve our coverage of the whole brain. Except for the striatum, we only selected papers on mice experiments. We also made sure to select a single paper per experiment to prevent any duplicate referencing. We extracted the mean value provided in each paper and its standard deviation whenever this value was available, Standard Error of the Mean were converted to standard deviations based on the numbers of individuals used to make the estimates. A density estimate in a specific brain region was assumed to be valid for the entire region. If the estimates were provided in the form of counts, we converted these into densities using the region volume from the paper, if available, or computed from the AV. When ratios (or percentages) of neuron type in a region were provided as proportions of the local cells or neurons populations, these ratios were multiplied by the corresponding densities from the BBCAv1 [5]. In total, we extracted density values from 54 different papers [2,3,5–56]. Our literature review is compiling the work of many papers. Any use of the results presented in this review should give the credit to the corresponding paper these results were extracted from. In particular, the major contributor of this review is the Kim et al. [6] study, and it should get a particular attention when our work is cited.

For each paper providing regional counts, densities, or proportion of inhibitory neuron types, we assigned the closest matching AV region(s). Sometimes several regions were assigned to the same literature value, while sometimes multiple values from literature were averaged to fit into a region of the AV. For instance, the cortical layer 6 is subdivided into two sublayers a and b in the AV but some papers do not make this distinction [7,52]. For these cases, we assumed the same densities for both subregions.

Several papers provided densities for the frontal cortex [50–52]. This region was assumed to cover the prelimbic area, the frontal pole and infralimbic area of the AV.

Almási et al. [7] provided inhibitory neuron densities in the somatosensory cortex, barrel field. The estimates from the layer 5a and 5b in the paper were averaged as the AV does not have this layer subdivision.

Bjerke et al. [9] stored their PV+ neuron density values in their supplementary Table 1.

From Fasulo et al. [11], we obtained PV+ neuron densities from their Figure 1CE.

Fazzari et al.’s counts of inhibitory neurons in 40 μm coronal slices of the isocortex [12] were extracted from the paper’s Figure 1.

We extracted PV+ neuron densities from the Figure 7 of Förster [13].

Gonchar et al. reported densities that we extracted from their Figure 2 [14].

In Gotts et al. [15], GAD67 immunoreactive neurons were counted in 50 μm sections of the nucleus of the solitary tract of the mouse brain (see their Table 1). We converted these counts into densities, using the mean volume of 50 μm coronal slices of the nucleus of the solitary tract in the AV. The paper’s estimates in the subregions of the nucleus of the solitary tract were matched with their closest region in the AV:

- Medial part (paper) 🡪 Gelatinous part (AV)
- Central part 🡪 Central part
- Commissural part 🡪 Commissural part
- Dorsomedial, Intermediate, Medial parts 🡪 Medial part
- Interstitial, Lateral, Ventrolateral, Ventral parts 🡪 Lateral part

We took the sum of the left and right hemisphere as this distinction does not exist in the AV. We also averaged all values assigned to the lateral and medial subregions in the AV.

The globus pallidus densities in Gourfinkel-An et al. [16] were assumed to be the same in both its internal and external segment subdivisions in the AV. A global estimate of inhibitory neurons in the entire cerebellar cortex was estimated from Gourfinkel-An et al. based on the authors estimates in the molecular and granular layers. These estimates were multiplied by the proportion of each layer volume in the cerebellar cortex. No voxels of the AV are labeled as belonging to the Purkinje Layer so its contribution to the cerebellar cortex density estimate was null.

Grünewald et al. [17] supplementary Table 2 lists all the interneuron densities reported by the authors.

Hafner et al. [18] provided counts of VIP+ neurons in the somatosensory cortex, barrel field. These counts were converted into densities using the size of the area used to count the cells (0.1 mm * 0.24 mm) multiplied by the mean depth of the region, based on measurements from Lefort et al. [24].

Han et al. [19] provided counts of GAD67 neurons from 30 μm‐thick coronal slices of the whole mouse brain. The mean surface occupied by the regions of the article in the coronal slices were estimated from similar coronal slices in the AV. The resulting densities are very low when compared to other literature sources. These values were therefore not considered in the fitting process.

Irintchev et al. [21] reported inhibitory neuron densities which we extracted from their Fig. 3. However, it was not very clear from which region of the somatosensory cortex one of their estimates were taken from. Hence, we considered these estimates for the entire region.

Jinno and Kosaka [22] and Whissell et al. [51] reported densities in the dorsal and ventral parts of the hippocampus. This subdivision of the hippocampus is not present in the AV, and as a result, the values reports were averaged to represent the full region. Additionally, estimates from the lateral and medial subdivisions of the visual areas from Whissell et al. were averaged for similar reasons.

Lefort et al. [24] provided counts of inhibitory neurons in a cylinder of the C2 barrel column of the mouse isocortex. We transformed the counts into densities based on the dimensions of the cylinder provided by the authors. We additionally applied the densities in the C2 barrel column to the whole barrel region (including the septa).

Moreno-Gonzalez et al. [26] provided densities of neurons in the Entorhinal area in the paper Figure 1.

We extracted manually the densities from the Figure 11 in Neddens and Buonanno [27].

In Okada et al. [29], densities of GAD67 neurons in the mouse nucleus of the solitary tract were measured by the authors in 4 areas of the regions. The average of the densities in the areas was taken, as the areas taken by the authors did not correspond to any of the AV subregions.

Ono et al. [30] provided densities of GABAergic neurons in cells / 10^4^ μm^2^ from 50 μm‐thick transverse slices of the mouse brain. No standard deviation value was provided in the paper.

Parrish-Aungust et al. [31] obtained multiple counts of inhibitory cells in their Table 4 that were converted into densities using the volumes of the regions provided by the authors in Table 2.

In Pirone et al. [32], the authors reported densities of inhibitory neurons in coronal sections from the Infralimbic and Prelimbic areas. We converted these densities in cells/mm^3^ based on the thickness of the sections (20 μm).

Pitts et al. [33] reported densities of cells/mm^2^ in 40 μm-thick slices in their Figure 3B.

Prönneke et al. [34] have reports of VIP neuron densities in the somatosensory cortex, barrel field.

From Ramos et al. [36], we extracted densities of SST+ neurons in different regions of the hippocampus from their Figure 2E.

In the Figure 8, of Ransome and Turnley [37], we could obtain densities from the Striatum.

Sanchez-Meijas et al. [38] reported densities of PV+ neurons from two regions of the cortex. We considered the Zone 35, and Zone 36 (in the paper) as respectively perirhinal area and the ectorhinal area of the isocortex.

Schmalbach et al. [39] have neuron densities reported that we extracted from their Figure 1 and 2.

Schmid et al [40] provided densities in cell/mm^2^ from 50 μm-thick slices in their Figure 1i.

Song et al. [42] provided counts of PV+ neurons (see their Table 1) in the striatum together with the volume in which the counting was performed.

We extracted density values from the Table 2 of Suzuki and Bekkers [43] in the anterior part of the piriform area of the olfactory areas (i.e. the part in contact with the lateral olfactory tracts). In absence of a better We assumed these estimates to be true in the entire piriform area, The layer 1, 2 and 3 were respectively associated to the molecular layer, the pyramidal layer and the polymorph layer. The a and b subdivisions of the layers 1 and 2 were not present in the AV so we averaged the two estimates for each layer.

Tamamaki et al. provided percentages of GABAergic cells among the population of neurons in regions of the isocortex [44]. We converted these proportions into densities using the neuron densities from the Cell Atlas.

A proportion of GABAergic neuron densities in the striatum were estimated from a collection of papers on the rodent [57–62], see review from Tepper et al. [45]. From these papers, we could estimate the total proportion of inhibitory neuron in the striatum as the sum of the proportions of each of its distinct GABAergic cell type:

- Medium spiny cells: 95% [57].
- Cholinergic: 1.7% [58].
- Parvalbumin: 0.7% [59,60].
- Calretinin: 0.8% [59].
- Neuropeptide Y: 0.9% [61].
- Tyrosine hydroxylase: 0.2% [62].

The total percent of inhibitory neuron in the striatum is therefore at least equal to 99.3%, which is the value we used in our pipeline.

We extracted PV and SST neuron densities from the main text and the Figure 1 of the paper of Trujilo-Estrada et al. [46].

We extracted from Figures 3 and 4 of Waider et al. [47] densities of PV expressing neurons.

Counts of GAD67 neurons in the mouse nucleus of the solitary tract from 10 sections (100 * 100 * 50 μm) were manually extracted from the Fig. 1F in Wang and Bradley [49].

Wang et al. [50] reported percent of inhibitory neuron cells according to the total cell population in their Figure 4. We used the BBCAV1 cell densities to convert this proportion into densities.

Xu et al. [52] reported densities in the layer 4 of the frontal cortex. But this layer does not appear in the frontal part of the cortex of the AV, which is why the value was ignored.

We extracted counts of PV+ neurons in the striatum from Yalcin-Cakmakli et al. [53]. To convert these counts into densities we used the volume of the striatum from the AV2a.

From the paper of Zhang et al. [55] we extracted the density values present in their Figure 6.

From Zhao et al. [56] Fig. 3 we manually extracted the counts and standard deviation of GAD67 positive cells in coronal slices of the lateral septal nucleus. The subregions from the paper are more precise than the AV. We averaged the intermediate counts values with the rostro-ventral counts and assigned them to the rostroventral region of the AV. The caudal-ventral counts from the paper were assigned to the ventral part of the AV as the voxels assigned to this region are located at the most caudal part of the lateral septal nucleus. The values left of the paper were averaged and assigned to the caudal-dorsal part of the AV. Counts were then converted to densities based on the volume of the counting frame (i.e., 163 * 163 * 30 µm).

From Erö et al. [5], we finally extracted the neuron densities in regions reported in literature to be fully inhibitory. These includes the layer 1 of the isocortex [35], the molecular layer of the cerebellar cortex [3], the reticular nucleus of the thalamus [20].

## **Finding an initial solution for the optimization**

After obtaining first estimates of counts, for each inhibitory neuron type, in each region of the brain, from the literature or the fitting, we need to test and correct them using the assumptions defined in the Assumptions section. There are two consequences of assumptions 2 and 3: First, the number of GAD67 positive neurons in each brain region must be smaller than the total number of neurons (computed at step 2 of the BBCAv2 pipeline - see Fig 1) and second, the sum of VIP, SST and PV positive neurons must also be smaller than the total number of neurons. Thus, whenever these conditions are violated, we scale down the number of these neurons while preserving their ratios (see Algorithm 1 in S2 Fig). This correction is applied in each region starting from leaf regions in the AV hierarchy to the top-level brain regions. Leaf regions are independent so corrections can be applied directly. For regions higher up in the hierarchy (e.g., barrel cortex), the estimated cell counts must equal the sum of the cell counts of their subregions (e.g., layers of barrel cortex). Solving this constraint is therefore easier following this order.

Then, we assert that our inhibitory neuron density estimates are taking into account our 4th assumption which states that PV, SST and VIP also express GAD67 and allows us to estimate nRest. Thus, for each region of the brain following the same order as described previously, whenever the constraint is violated, we scale down the number of the PV+ SST+ and VIP+ neurons while preserving their relative ratios and conversely increase the number of GAD67+ neurons (see Algorithm 2 in S3 Fig).

Algorithms 1 and 2 are applied to each region of the AV starting from leaf regions in the hierarchy tree to the major brain regions. This implies that the corrections applied to a region are impacted by the ones applied on its subregions but not the other way around. Hence, these algorithms are appropriate to find a good initial solution for the Cell Atlas model (see combination section) but does not guarantee an optimal result, i.e., as closed as possible from the literature and the fitting original estimates.

# Supplementary Tables

| **Abbreviation** | **Full name** |
| --- | --- |
| AAV | Adeno-associated virus |
| ACA | Anterior Cingulate Area |
| ACAd | Anterior cingulate area, dorsal part |
| ACAv | Anterior cingulate area, ventral part |
| AIBS | Allen Institute for Brain Science |
| AId | Agranular insular area, dorsal part |
| AIp | Agranular insular area, posterior part |
| AIv | Agranular insular area, ventral part |
| AUDd | Dorsal auditory area |
| AUDp | primary auditory cortex |
| AUDpo | Posterior auditory area |
| AUDv | Ventral auditory area |
| AV | Annotation Atlas Volume |
| BBCA | Blue Brain Mouse Cell Atlas pipeline |
| BF / SSp-bfd | Primary somatosensory area, barrel field |
| CA1 | Field CA1 |
| CA3 | Field CA3 |
| CBXpu | CereBellar corteX, Purkinje layer |
| CCF | Common Coordinate Framework |
| DG | Dentate Gyrus |
| ECT | Ectorhinal area |
| EXC | excitatory neurons |
| FRP | Frontal pole, cerebral cortex |
| GABA | gamma-Aminobutyric acid |
| GAD | glutamic acid decarboxylase |
| GU | Gustatory areas |
| ILA | Infralimbic area |
| INH | inhibitory neuron |
| ISH | *in situ* hybridization |
| LAMP5 | lysosomal associated membrane protein family member 5 |
| MOp | Primary motor area |
| MOs | Secondary motor area |
| ORBl | Orbital area, lateral part |
| ORBm | Orbital area, medial part |
| ORBvl | Orbital area, ventrolateral part |
| PERI | Perirhinal area |
| PL | PreLimbic area |
| PTLp | Posterior parietal association areas |
| PV | parvalbumin |
| RSPagl | Retrosplenial area, lateral agranular part |
| RSPd | Retrosplenial area, dorsal part |
| RSPv | Retrosplenial area, ventral part |
| SS / SSp | Primary SomatoSensory area |
| SSp-ll | primary somatosensory cortex lower limb |
| SSp-m | Primary somatosensory area, mouth |
| SSp-n | Primary somatosensory area, nose |
| SSp-tr | Primary somatosensory area, trunk |
| SSs | Supplemental somatosensory area |
| SST | somatostatin |
| STR | STRiatum |
| SUB | SUBiculum |
| TEa | temporal association areas |
| VIP | vasoactive intestinal peptide |
| VISal | Anterolateral visual area |
| VISam | Anteromedial visual area |
| VISC | Visceral area |
| VISl | Lateral visual area |
| VISp / VA | Primary VISual area |
| VISpl | Posterolateral visual area |
| VISpm | Posteromedial visual area |

## **Table A. Nonstandard abbreviations**

List of all abbreviations used in this study. Region abbreviations are related to Figs 4 and 8.

# References

1. Markram H, Muller E, Ramaswamy S, Reimann MW, Abdellah M, Sanchez CA, et al. Reconstruction and Simulation of Neocortical Microcircuitry. Cell. 2015;163: 456–492. doi:10.1016/j.cell.2015.09.029

2. Jinno S, Aika Y, Fukuda T, Kosaka T. Quantitative analysis of GABAergic neurons in the mouse hippocampus, with optical disector using confocal laser scanning microscope. Brain Res. 1998;814: 55–70. doi:10.1016/S0006-8993(98)01075-0

3. Casali S, Marenzi E, Medini C, Casellato C, D’Angelo E. Reconstruction and Simulation of a Scaffold Model of the Cerebellar Network. Front Neuroinformatics. 2019;13. doi:10.3389/fninf.2019.00037

4. Meyer HS, Schwarz D, Wimmer VC, Schmitt AC, Kerr JND, Sakmann B, et al. Inhibitory interneurons in a cortical column form hot zones of inhibition in layers 2 and 5A. Proc Natl Acad Sci. 2011;108: 16807–16812. doi:10.1073/pnas.1113648108

5. Erö C, Gewaltig M-O, Keller D, Markram H. A Cell Atlas for the Mouse Brain. Front Neuroinformatics. 2018;12. doi:10.3389/fninf.2018.00084

6. Kim Y, Yang GR, Pradhan K, Venkataraju KU, Bota M, García del Molino LC, et al. Brain-wide Maps Reveal Stereotyped Cell-Type-Based Cortical Architecture and Subcortical Sexual Dimorphism. Cell. 2017;171: 456-469.e22. doi:10.1016/j.cell.2017.09.020

7. Almási Z, Dávid C, Witte M, Staiger JF. Distribution Patterns of Three Molecularly Defined Classes of GABAergic Neurons Across Columnar Compartments in Mouse Barrel Cortex. Front Neuroanat. 2019;13. doi:10.3389/fnana.2019.00045

8. Arcelli P, Frassoni C, Regondi MC, Biasi SD, Spreafico R. GABAergic Neurons in Mammalian Thalamus: A Marker of Thalamic Complexity? Brain Res Bull. 1997;42: 27–37. doi:10.1016/S0361-9230(96)00107-4

9. Bjerke IE, Yates SC, Laja A, Witter MP, Puchades MA, Bjaalie JG, et al. Densities and numbers of calbindin and parvalbumin positive neurons across the rat and mouse brain. iScience. 2021;24. doi:10.1016/j.isci.2020.101906

10. Calfa G, Li W, Rutherford JM, Pozzo-Miller L. Excitation/Inhibition Imbalance and Impaired Synaptic Inhibition in Hippocampal Area CA3 of Mecp2 Knockout Mice. Hippocampus. 2015;25: 159–168. doi:10.1002/hipo.22360

11. Fasulo L, Brandi R, Arisi I, La Regina F, Berretta N, Capsoni S, et al. ProNGF Drives Localized and Cell Selective Parvalbumin Interneuron and Perineuronal Net Depletion in the Dentate Gyrus of Transgenic Mice. Front Mol Neurosci. 2017;10. doi:10.3389/fnmol.2017.00020

12. Fazzari P, Mortimer N, Yabut O, Vogt D, Pla R. Cortical distribution of GABAergic interneurons is determined by migration time and brain size. Development. 2020;147: dev185033. doi:10.1242/dev.185033

13. Förster JA. Quantitative morphological analysis of the neostriatum and the cerebellum of tenascin-C deficient mice. Quantitative morphologische Analysen des Neostriatums und des Cerebellums der Tenascin-C defizienten Maus. 2008 [cited 18 Jan 2021]. Available: https://ediss.sub.uni-hamburg.de/handle/ediss/2354

14. Gonchar Y, Wang Q, Burkhalter AH. Multiple distinct subtypes of GABAergic neurons in mouse visual cortex identified by triple immunostaining. Front Neuroanat. 2008;2. doi:10.3389/neuro.05.003.2007

15. Gotts J, Atkinson L, Edwards IJ, Yanagawa Y, Deuchars SA, Deuchars J. Co-expression of GAD67 and choline acetyltransferase reveals a novel neuronal phenotype in the mouse medulla oblongata. Auton Neurosci. 2015;193: 22–30. doi:10.1016/j.autneu.2015.05.003

16. Gourfinkel‐An I, Parain K, Hartmann A, Mangiarini L, Brice A, Bates G, et al. Changes in GAD67 mRNA expression evidenced by in situ hybridization in the brain of R6/2 transgenic mice. J Neurochem. 2003;86: 1369–1378. doi:https://doi.org/10.1046/j.1471-4159.2003.01916.x

17. Grünewald B, Lange MD, Werner C, O’Leary A, Weishaupt A, Popp S, et al. Defective synaptic transmission causes disease signs in a mouse model of juvenile neuronal ceroid lipofuscinosis. Rosenmund C, editor. eLife. 2017;6: e28685. doi:10.7554/eLife.28685

18. Hafner G, Guy J, Witte M, Truschow P, Rüppel A, Sirmpilatze N, et al. Circuits in the absence of cortical layers: increased callosal connectivity in reeler mice revealed by brain-wide input mapping of VIP neurons in barrel cortex. bioRxiv. 2020; 2020.04.19.048868. doi:10.1101/2020.04.19.048868

19. Han L-C, Zhang H, Wang W, Wei Y-Y, Sun X-X, Yanagawa Y, et al. The Effect of Sevoflurane Inhalation on Gabaergic Neurons Activation: Observation on the GAD67-GFP Knock-In Mouse. Anat Rec Adv Integr Anat Evol Biol. 2010;293: 2114–2122. doi:10.1002/ar.21113

20. Hou G, Smith AG, Zhang Z-W. Lack of Intrinsic GABAergic Connections in the Thalamic Reticular Nucleus of the Mouse. J Neurosci. 2016;36: 7246–7252. doi:10.1523/JNEUROSCI.0607-16.2016

21. Irintchev A, Rollenhagen A, Troncoso E, Kiss JZ, Schachner M. Structural and Functional Aberrations in the Cerebral Cortex of Tenascin-C Deficient Mice. Cereb Cortex. 2005;15: 950–962. doi:10.1093/cercor/bhh195

22. Jinno S, Kosaka T. Cellular architecture of the mouse hippocampus: A quantitative aspect of chemically defined GABAergic neurons with stereology. Neurosci Res. 2006;56: 229–245. doi:10.1016/j.neures.2006.07.007

23. Lange W. Cell number and cell density in the cerebellar cortex of man and some other mammals. Cell Tissue Res. 1975;157: 115–124. doi:10.1007/BF00223234

24. Lefort S, Tomm C, Floyd Sarria J-C, Petersen CCH. The Excitatory Neuronal Network of the C2 Barrel Column in Mouse Primary Somatosensory Cortex. Neuron. 2009;61: 301–316. doi:10.1016/j.neuron.2008.12.020

25. Leitner FC, Melzer S, Lütcke H, Pinna R, Seeburg PH, Helmchen F, et al. Spatially segregated feedforward and feedback neurons support differential odor processing in the lateral entorhinal cortex. Nat Neurosci. 2016;19: 935–944. doi:10.1038/nn.4303

26. Moreno-Gonzalez I, Baglietto-Vargas D, Sanchez-Varo R, Jimenez S, Trujillo-Estrada L, Sanchez-Mejias E, et al. Extracellular Amyloid-β and Cytotoxic Glial Activation Induce Significant Entorhinal Neuron Loss in Young PS1M146L/APP751SL Mice. J Alzheimers Dis. 2009;18: 755–776. doi:10.3233/JAD-2009-1192

27. Neddens J, Buonanno A. Selective populations of hippocampal interneurons express ErbB4 and their number and distribution is altered in ErbB4 knockout mice. Hippocampus. 2010;20: 724–744. doi:https://doi.org/10.1002/hipo.20675

28. Nirgudkar P, Taylor DH, Yanagawa Y, Valenzuela CF. Ethanol exposure during development reduces GABAergic/glycinergic neuron numbers and lobule volumes in the mouse cerebellar vermis. Neurosci Lett. 2016;632: 86–91. doi:10.1016/j.neulet.2016.08.039

29. Okada T, Tashiro Y, Kato F, Yanagawa Y, Obata K, Kawai Y. Quantitative and immunohistochemical analysis of neuronal types in the mouse caudal nucleus tractus solitarius: Focus on GABAergic neurons. J Chem Neuroanat. 2008;35: 275–284. doi:10.1016/j.jchemneu.2008.02.001

30. Ono M, Yanagawa Y, Koyano K. GABAergic neurons in inferior colliculus of the GAD67-GFP knock-in mouse: Electrophysiological and morphological properties. Neurosci Res. 2005;51: 475–492. doi:10.1016/j.neures.2004.12.019

31. Parrish-Aungst S, Shipley MT, Erdelyi F, Szabo G, Puche AC. Quantitative analysis of neuronal diversity in the mouse olfactory bulb. J Comp Neurol. 2007;501: 825–836. doi:10.1002/cne.21205

32. Pirone A, Alexander JM, Koenig JB, Cook-Snyder DR, Palnati M, Wickham RJ, et al. Social Stimulus Causes Aberrant Activation of the Medial Prefrontal Cortex in a Mouse Model With Autism-Like Behaviors. Front Synaptic Neurosci. 2018;10: 35. doi:10.3389/fnsyn.2018.00035

33. Pitts MW, Reeves MA, Hashimoto AC, Ogawa A, Kremer P, Seale LA, et al. Deletion of Selenoprotein M Leads to Obesity without Cognitive Deficits *. J Biol Chem. 2013;288: 26121–26134. doi:10.1074/jbc.M113.471235

34. Prönneke A, Scheuer B, Wagener RJ, Möck M, Witte M, Staiger JF. Characterizing VIP Neurons in the Barrel Cortex of VIPcre/tdTomato Mice Reveals Layer-Specific Differences. Cereb Cortex. 2015;25: 4854–4868. doi:10.1093/cercor/bhv202

35. Ramaswamy S, Markram H. Anatomy and physiology of the thick-tufted layer 5 pyramidal neuron. Front Cell Neurosci. 2015;9: 233. doi:10.3389/fncel.2015.00233

36. Ramos B, Baglietto-Vargas D, Rio JC del, Moreno-Gonzalez I, Santa-Maria C, Jimenez S, et al. Early neuropathology of somatostatin/NPY GABAergic cells in the hippocampus of a PS1×APP transgenic model of Alzheimer’s disease. Neurobiol Aging. 2006;27: 1658–1672. doi:10.1016/j.neurobiolaging.2005.09.022

37. Ransome MI, Turnley AM. Analysis of neuronal subpopulations in mice over-expressing suppressor of cytokine signaling-2. Neuroscience. 2005;132: 673–687. doi:10.1016/j.neuroscience.2004.12.041

38. Sanchez‐Mejias E, Nuñez‐Diaz C, Sanchez‐Varo R, Gomez‐Arboledas A, Garcia‐Leon JA, Fernandez‐Valenzuela JJ, et al. Distinct disease‐sensitive GABAergic neurons in the perirhinal cortex of Alzheimer’s mice and patients. Brain Pathol. 2020;30: 345–363. doi:10.1111/bpa.12785

39. Schmalbach B, Lepsveridze E, Djogo N, Papashvili G, Kuang F, Leshchyns’ka I, et al. Age-dependent loss of parvalbumin-expressing hippocampal interneurons in mice deficient in CHL1, a mental retardation and schizophrenia susceptibility gene. J Neurochem. 2015;135: 830–844. doi:https://doi.org/10.1111/jnc.13284

40. Schmid JS, Bernreuther C, Nikonenko AG, Ling Z, Mies G, Hossmann K-A, et al. Heterozygosity for the mutated X-chromosome-linked L1 cell adhesion molecule gene leads to increased numbers of neurons and enhanced metabolism in the forebrain of female carrier mice. Brain Struct Funct. 2013;218: 1375–1390. doi:10.1007/s00429-012-0463-9

41. Seabrook TA, Krahe TE, Govindaiah G, Guido W. Interneurons in the mouse visual thalamus maintain a high degree of retinal convergence throughout postnatal development. Neural Develop. 2013;8: 24. doi:10.1186/1749-8104-8-24

42. Song C-H, Bernhard D, Bolarinwa C, Hess EJ, Smith Y, Jinnah HA. Subtle microstructural changes of the striatum in a DYT1 knock-in mouse model of dystonia. Neurobiol Dis. 2013;54: 362–371. doi:10.1016/j.nbd.2013.01.008

43. Suzuki N, Bekkers JM. Inhibitory neurons in the anterior piriform cortex of the mouse: Classification using molecular markers. J Comp Neurol. 2010;518: 1670–1687. doi:10.1002/cne.22295

44. Tamamaki N, Yanagawa Y, Tomioka R, Miyazaki J-I, Obata K, Kaneko T. Green fluorescent protein expression and colocalization with calretinin, parvalbumin, and somatostatin in the GAD67-GFP knock-in mouse. J Comp Neurol. 2003;467: 60–79. doi:10.1002/cne.10905

45. Tepper J, Tecuapetla F, Koos T, Ibanez-Sandoval O. Heterogeneity and Diversity of Striatal GABAergic Interneurons. Front Neuroanat. 2010;4. Available: https://www.frontiersin.org/article/10.3389/fnana.2010.00150

46. Trujillo-Estrada L, Dávila JC, Sánchez-Mejias E, Sánchez-Varo R, Gomez-Arboledas A, Vizuete M, et al. Early Neuronal Loss and Axonal/Presynaptic Damage is Associated with Accelerated Amyloid-β Accumulation in AβPP/PS1 Alzheimer’s Disease Mice Subiculum. J Alzheimers Dis. 2014;42: 521–541. doi:10.3233/JAD-140495

47. Waider J, Proft F, Langlhofer G, Asan E, Lesch K-P, Gutknecht L. GABA concentration and GABAergic neuron populations in limbic areas are differentially altered by brain serotonin deficiency in Tph2 knockout mice. Histochem Cell Biol. 2013;139: 267–281. doi:10.1007/s00418-012-1029-x

48. Wall NR, De La Parra M, Sorokin JM, Taniguchi H, Huang ZJ, Callaway EM. Brain-Wide Maps of Synaptic Input to Cortical Interneurons. J Neurosci. 2016;36: 4000–4009. doi:10.1523/JNEUROSCI.3967-15.2016

49. Wang M, Bradley RM. Properties of GABAergic Neurons in the Rostral Solitary Tract Nucleus in Mice. J Neurophysiol. 2010;103: 3205–3218. doi:10.1152/jn.00971.2009

50. Wang X, Allen WE, Wright MA, Sylwestrak EL, Samusik N, Vesuna S, et al. Three-dimensional intact-tissue sequencing of single-cell transcriptional states. Science. 2018;361: eaat5691. doi:10.1126/science.aat5691

51. Whissell PD, Cajanding JD, Fogel N, Kim JC. Comparative density of CCK- and PV-GABA cells within the cortex and hippocampus. Front Neuroanat. 2015;9. doi:10.3389/fnana.2015.00124

52. Xu X, Roby KD, Callaway EM. Immunochemical characterization of inhibitory mouse cortical neurons: Three chemically distinct classes of inhibitory cells. J Comp Neurol. 2010;518: 389–404. doi:10.1002/cne.22229

53. Yalcin-Cakmakli G, Rose SJ, Villalba RM, Williams L, Jinnah HA, Hess EJ, et al. Striatal Cholinergic Interneurons in a Knock-in Mouse Model of L-DOPA-Responsive Dystonia. Front Syst Neurosci. 2018;12. Available: https://www.frontiersin.org/articles/10.3389/fnsys.2018.00028

54. Yamanaka H, Yanagawa Y, Obata K. Development of stellate and basket cells and their apoptosis in mouse cerebellar cortex. Neurosci Res. 2004;50: 13–22. doi:10.1016/j.neures.2004.06.008

55. Zhang C, Yan C, Ren M, Li A, Quan T, Gong H, et al. A platform for stereological quantitative analysis of the brain-wide distribution of type-specific neurons. Sci Rep. 2017;7: 14334. doi:10.1038/s41598-017-14699-w

56. Zhao C, Eisinger B, Gammie SC. Characterization of GABAergic Neurons in the Mouse Lateral Septum: A Double Fluorescence In Situ Hybridization and Immunohistochemical Study Using Tyramide Signal Amplification. Fatemi H, editor. PLoS ONE. 2013;8: e73750. doi:10.1371/journal.pone.0073750

57. Gerfen CR, Wilson CJ. Chapter II The basal ganglia. Handbook of Chemical Neuroanatomy. Elsevier; 1996. pp. 371–468. doi:10.1016/S0924-8196(96)80004-2

58. Phelps PE, Houser CR, Vaughn JE. Immunocytochemical localization of choline acetyltransferase within the rat neostriatum: A correlated light and electron microscopic study of cholinergic neurons and synapses. J Comp Neurol. 1985;238: 286–307. doi:10.1002/cne.902380305

59. Rymar VV, Sasseville R, Luk KC, Sadikot AF. Neurogenesis and stereological morphometry of calretinin-immunoreactive GABAergic interneurons of the neostriatum. J Comp Neurol. 2004;469: 325–339. doi:10.1002/cne.11008

60. Luk KC, Sadikot AF. GABA promotes survival but not proliferation of parvalbumin-immunoreactive interneurons in rodent neostriatum: an in vivo study with stereology. Neuroscience. 2001;104: 93–103. doi:10.1016/S0306-4522(01)00038-0

61. Ibáñez-Sandoval O, Tecuapetla F, Unal B, Shah F, Koós T, Tepper JM. A Novel Functionally Distinct Subtype of Striatal Neuropeptide Y Interneuron. J Neurosci. 2011;31: 16757–16769. doi:10.1523/JNEUROSCI.2628-11.2011

62. Ünal B, Shah F, Kothari J, Tepper JM. Anatomical and electrophysiological changes in striatal TH interneurons after loss of the nigrostriatal dopaminergic pathway. Brain Struct Funct. 2015;220: 331–349. doi:10.1007/s00429-013-0658-8
