# Supplementary material for: A method to estimate the cellular composition of the mouse brain from heterogeneous datasets
Source: PLoS Comput Biol. 2022 Dec 21;18(12):e1010739. doi: 10.1371/journal.pcbi.1010739 (PMC9838873; doi:10.1371/journal.pcbi.1010739)
Supplement: Attachment — Submitted filename: Responses_to_reviewers.pdf [file pcbi.1010739.s013.pdf]

August 15th, 2022

Subject: Revision and resubmission of manuscript PCOMPBIOL-D-22-00098

Dear Editor in chief,

Please find attached a re-submission of the revised manuscript entitled: “A method to estimate the cellular composition of the mouse brain from heterogeneous datasets”, as a full paper. We wish to thank all the Reviewers for their constructive and very helpful suggestions. We kindly provide our responses to the Reviewers’ comments and believe that our revised manuscript has been improved and may now be suitable for publication in *Plos Computational Biology*. The modifications have been highlighted in green in the final manuscript.

Reviewers’ comments:

Reviewer #1:

MAJOR:

- Cell density estimations:

1. The authors use a binary thresholding method on ISH images and then attempt to use the intensity values and literature data to predict cell densities in regions where no literature is available.
The core assumption, as stated in section 2.1, is that there is "a correlation between genetic marker expression and cell type density gathered from the literature and the previously aligned and filtered ISH datasets".
However, this assumption is shown by the authors not to be true, as evidenced by the poor performance of the transfer function.

OUR RESPONSE: The reviewer rightfully points out that Fig. 5 depicts an imperfect fit and suggests that our assumption on ISH datasets is incorrect.
Our assumption states that, in a brain region, the mean region intensity, defined as the average number of voxels positive to ISH (i.e., where cells expressing the marker were detected) in these images correlates with the density of cells expressing the marker in this region. This correlation is further supported when computing the Pearson correlation coefficient (ρ=0.59) between the extracted mean region intensities and corresponding literature density values (for all markers). To further support our hypothesis, we produced a new linear fitting (see new supplementary S9 Fig.) between the literature values altogether and the corresponding mean intensities. S9 Fig.A shows indeed a clear relationship between literature values and region mean intensity, also supporting

our assumption. We also observe a wide spread of the points, especially for low values). As we have demonstrated in length in our manuscript there is a large variability in the cell densities reported by literature (see Fig. 4B). This explains the spreading of the points along the y axis in S9 Fig.A (different point colors).This variability cannot be solved with any change of method to extract the x-axis values.

We use the relationship described in our assumption to construct transfer functions linking densities of GAD67+, PV+, SST+, and VIP+ cells reported in the literature to the mean intensity in each region of the AV. As we are leveraging every individual literature value obtained through our review, we cannot expect to obtain a perfect linear fit. For the point cloud of S9 Fig.A, we calculated the coefficient of determination of the fit (R^2). R^2= 34%, which means that the great variability of the point cloud could not be fully accounted for. We therefore improve the quality of our fitting by computing separately a transfer function for each of our genetic markers (see S9 Fig. B, different colors indicate the different markers) and by furthermore splitting the fitting by groups of regions (here, isocortex, cerebellum and rest. See Figure 5). The overall quality of the transfer functions has overall improved with the splitting into subgroups. This splitting can further be refined at the next iterations of the pipeline.

We rewrote Section 2.6 to explain with more details how we justify our assumption and how the quality of the fitting improved with the different splitting of the data.

The authors show that there is no significant improvement when averaging two datasets for a marker.

OUR RESPONSE: We show indeed that we did not improve the quality of the fit using the combination of two ISH datasets from the AIBS. However, as we described in our previous answer, changing the method to extract ISH data will only impact the x axis of Fig. 5. The spreading of points along the y-axis will not be resolved (e.g., when multiple literature sources reporting values in the same region contradict each other). We added this fact to Section 3.4 to support this point further. Additionally, as we describe in Section , this integration might have not significantly improved the coverage of the brain or resolved potential remaining artifacts in the binarized ISH images.

The qualitative validation (Fig. 10) further shows major differences between the real and the predicted sagittal data.

OUR RESPONSE: In Figure 10AB, we compared a real GAD67 ISH slice with its virtual slice counterpart from the BBCAv2. As discussed in the Sections 3.4 and 4.8, the virtual slice of the BBCAv2 does not take into account the size of the somas and their placement within a voxel are random, which means that we do not check their overlapping. Moreover, the tips of the brain are cut in the AV but not in the real slice. Finally, the GAD67 sagittal slice was not realigned to our model which means that its shape might differ slightly from our brain volume.

Taking all of these details into account, we noted some differences that could not be explained by the previous points and discussed each of them. In the case of the hippocampus dentate gyrus, we provided a new complete analysis in Section 3.4 and an additional supplementary figure (S7. Fig) to explain the differences between our slice and the real one. For the cerebellum and the thalamus, we added a paragraph in Section 4.8. The difference observed in Figure 10AB might be linked to a population of inhibitory neurons in these regions both expressing the PV and SST gene. This statement is supported by the results of Zeisel et al. [1] and a manual inspection of

GAD67, PV and SST ISH slices. Our assumptions in this region should be therefore refined in these regions to take these specificities into account.

The differences observed in Fig. 10AB are therefore not due to our method but rather to some regional specificities that we will integrate in a future version of our model.

Given all of this, I find it curious that the authors do not choose another approach for estimating cell densities from the AIBS images.

They mention this opportunity in the discussion, but only as a future direction. I think it should be considered for the current paper, at least for the GAD67 dataset where literature data are so scarce, but preferably for all the markers.

OUR RESPONSE: We understand the reviewer concern regarding any potential improvement of our method. As we pointed out in a previous response, changing the method to extract cell density information from the ISH dataset would only partially resolve the issues of the fitting (i.e., only the x-axis of Figure 5. would be improved). But we have shown in Section 2.5 that the variability of the literature estimates is also quite important.

Erö et al. [2] considered different alternative methods and found that, in many brain regions, evaluating ISH marker intensity yields better results than using cell counting, because of factors such as cell overlap (see Figure 1 and Figure 7 in Erö et al.'s paper). Moreover, cell counting requires the realignment of the high-resolution images from the AIBS to the Nissl 25 μm and annotation volumes in order to delimit the regions of the brain in the image slice. The difference in resolution makes the realignment more difficult and the borders of the regions would be less precise due to the low resolution of the associated reference volumes. In addition, the cell counting methods are more sensitive to noise and artifacts that are still present in the ISH high resolution images. These facts were added to Section 4.3 to complete our discussion on cell counting techniques.

In this manuscript, we wanted to answer two questions of a previously published processing pipeline: First, how does the differences on annotation and reference volumes of various atlases affect the estimation of densities in a region and second, how can we integrate all available literature data, knowing that each individual estimate carries a considerable uncertainty? Changing other parts of the processing pipeline is beyond the scope of this manuscript. We updated both the introduction (Section 1) and the discussion (Section 4.3) to outline the scope of the paper more clearly.

2. The most important consequence of the above point is that the resulting density estimates are heavily biased by the method used to compute them. For the three interneuron subtypes for which data are presented in this study, brain-wide estimates are available from the literature (from Kim et al). In contrast, cell density estimates for all interneurons (assessed through GAD67 expression) are scarcely available. The main contribution then - in terms of new data - are the estimates of GAD67 and (by subtraction of the specific types above) the proportion of interneurons not expressing PV, SST or VIP. The finding that such a large proportion of GAD67 neurons are not expressing any of these

proteins is highly unexpected, as these cell types are typically considered to make up most of the interneuron population across the brain (Rudy, 2010).

OUR RESPONSE: It is true that in the somatosensory cortex, as reported by Lee, Rudy et al. [3], the PV, SST and VIP populations represent a large part of the inhibitory neuron population. Our results indeed indicate that this ratio might not apply to all regions in the brain. Furthermore, the striatum is a region almost if not entirely inhibitory (see review from Tepper et al. [4]). Among the inhibitory neuron population in this region, 95% of them are medium spiny neurons. This population has been shown by Zeisel et al. [1] to be expressing neither PV, nor SST nor VIP. In summary, our results are not quite as extraordinary as they may appear at first glance. We added a paragraph to discuss this point in Section 4.8.

However, given that the literature estimates for the GAD67 population covers so few brain regions compared to the other three cell types, these numbers are in >80 % of cases based on the intensity analysis and transfer function, which the authors quite clearly demonstrate does not correspond well with empirical data from the literature.

OUR RESPONSE: The first estimates of the GAD67 neuron densities are indeed mostly based on the transfer functions from marker intensity to literature density. We understand the reviewer's concern about the quality of the estimates obtained through the fitting. However, the fitting density estimates, as well as literature data density estimates are not the final values of the cell atlas. These estimates are further optimized to be integrated into our model, as described in Section 2.7. This means that the densities of PV, SST, VIP but also the densities of neurons from each region have an impact on the densities of GAD67. The final GAD67 density estimates are therefore not only based on the fitting. Moreover, we have shown that our algorithm could predict 10 % of the literature values from the 90% percent remaining values (see Figure 9B); therefore, leveraging the fitting.

A more refined method for extracting cell densities from the AIBS images (as mentioned in the comment above) would be needed to substantiate the claim that such a large proportion of GAD67 neurons do not express PV, SST or VIP. If it is indeed true, this is a very interesting finding that should be discussed further. However, without further confirmation I am inclined to believe that the incongruence between the PV+SST+VIP numbers and total GAD67 numbers are rather a byproduct of using very different methods to compute them.

OUR RESPONSE: Considering that our findings are not quite as extraordinary as they may appear (see above) and that we can explain a large fraction of the variability of the fit, we think that our results are substantiated. If any reviewed paper would contradict these results, our pipeline will be refined by integrating these findings. We added a paragraph in the discussion (Section 4.8) to further explain this proportion in the rest of the population in the brain.

3. While the integration of the Kim et al. data in the author's Cell Atlas is powerful, and the other estimations might be useful as a first approximation for computational models, the authors are making claims about these data that are not justified given the limitations and biases of their approach. For example, the claim in the introduction that "these refinements will help neuroscientists to get a better understanding at cellular composition" and the use of terms like "unbiased" and "coherent estimates".

OUR RESPONSE: We thank the reviewer for the concern about the way we describe our approach.

Our Cell Atlas model is the outcome of a data-driven approach that integrates all available literature findings on cellular composition in the mouse brain into a common framework. The model provides density estimates of various cell types in each region of the brain. Some of these estimates are based on the literature or inferred from literature and ISH datasets. Our results show that the pipeline is capable of predicting literature findings based on our assumptions (Figure 9B). On top of this, every density estimate obtained from literature, or the ISH dataset is integrated so that it does not break the internal composition rules of the model. In the end, our model therefore provides density estimates that are coherent with respect to each other. We believe our model can be therefore useful for the neuroscience community as it provides a coherent framework of cell density estimates in the mouse brain, even in regions for which no literature data is available. When modeling brain tissues, the basic approach is to choose one or more literature values to constrain the model. This choice is justified but introduces a bias towards the chosen values. Instead, we are leveraging available peer-reviewed literature sources on cellular counts regardless of their content. Thus, we consider our approach of literature integration unbiased.

We checked and added precision for each use of the terms listed by the reviewer (see Introduction Section).

- Choice of reference atlas and registration of data to it:

1. In the introduction, the authors state that "alignment of multiple datasets to a common coordinate system presents a significant source of uncertainty that we measured and addressed with deep learning techniques". I believe that the authors with "the source of uncertainty that we measured" are referring to their selection of brain reference atlas based on the resulting cell density distribution (Section 2.2). However, in this section they are measuring the fit of different combinations of the AIBS Nissl and annotation volumes. They do not address the uncertainty in alignment of multiple datasets (e.g., the ISH data used in their analysis).

OUR RESPONSE: We thank the reviewer for bringing our attention to this sentence where we were indeed not clear enough. We reformulated it to explain that we have measured the impact of misalignment of whole brain datasets on cell density estimations and that we therefore used a tool to automatically realign ISH datasets to the Nissl volume.

A notable source of uncertainty is that histological sections typically deviate from the standard plane, this is evident also in the AIBS ISH data (particularly in the somatostatin data used in the current study). It is not clear whether the authors' approach accounts for this. The authors should measure (or at least discuss) the uncertainty in their registration of the ISH data. They should also show images of the registration results for the ISH data so that the reader can qualitatively assess the fit.

OUR RESPONSE: This is indeed a point that was not developed in our paper. We considered the ISH slices to be perfectly vertical as the realignment tool that we used was trained with the same assumption and interpolation between nonparallel slices is more difficult. In a future version of the atlas, we will integrate the ISH slices considering their orientations. More discussion on the quality of the deep learning tool used to realign ISH slices is given in Krepl et al. [5]. We added these statements to the manuscript in Section 2.3 and 4.3 and added a supplementary figure to visualize the effect of realignment on the ISH slices.

2. Section 2.2 gives a summary of the different Nissl and annotation versions from the Allen Institute, which provides important background for the reader to understand the methodological decisions of the authors. However, I find this part to be inaccurate or wrong in several places, for example:

a. The authors do not mention the different versions of the annotation volume for CCFv3, and do not specify which one was used in their own analysis for selecting the reference atlas (Section 2.2).

OUR RESPONSE: We thank the reviewer for the comment. The version of the CCFv3 annotation atlas used in the manuscript corresponds to the 2017 version of the AIBS. This statement was added to the text.

b. According to the technical white papers from the AIBS, CCFv1 was published in 2005 and contained ~200 delineations. Clearly it is not this version that the authors have used in their analysis of the ISH data, since the number of brain regions in their AV is 955.

OUR RESPONSE: After investigation, the version of the reference volumes that was used by Erö et al. [2] is indeed not the CCFv1 but corresponds to an old version of the CCFv2, which seems to be no longer accessible on the AIBS website. This version does not have the ventricular systems labeling in the annotation volume. We removed the reference to CCFv1 and added this statement to Section 2.2

c. It is misleading to say that subregions are "merged" in the AV3, as all annotations were re-drawn in 3D for this version of the CCF

OUR RESPONSE: We agree with this point, and we have changed the term “merged” to “re-drawn”.

The authors should sort out these points and should also stay as close as possible to the terminology and versioning scheme used by the Allen Institute; currently there is some mixing of terms like "AV" and "AMBA".

OUR RESPONSE: We thank the reviewer for pointing out the term "AMBA" which was mistakenly used in the manuscript, we removed it. We understand the reviewer's concern about renaming the "Allen Mouse Brain Atlas" into "Annotations Volume", however we believe the AMBA terminology to be too similar to our BBCA terminology and could potentially confuse readers.

OTHER COMMENTS:

The methodology for the literature search is very scarcely described, providing no details on e.g., how, when, or through which search engine was performed nor of the search strings employed.

It is good that the supplementary file giving the data and details from the survey is included, but the file needs revision for clarity and overall organization. There are also some inconsistencies with the information provided in the text, e.g., the main text says there are 29 PV papers, but supplementary file only contains 23 references in total for PV, SST and VIP. In the result it is stated that they "integrated values from 56 different literature sources".

OUR RESPONSE: We added a paragraph in the supplementary materials to explain in detail our literature review method. As suggested later by the reviewer, we also added the complete list of papers collected during our literature review in the main text. We also made explicit for each neuron type from which papers we collected data. The excel sheet provided with our first version of the manuscript was outdated which explains the count difference observed by the reviewer. This excel file has also been updated.

In the discussion, they state that they perform a review of quantitative stereological studies, but several of the sources are not using stereology as the cell counting method.

OUR RESPONSE: We thank the reviewer for pointing out the misuse of the term stereological, we removed it from the discussion.

I believe all references from the literature search should be cited in the main text, as these papers have provided important data for the current study that should receive proper citation.

OUR RESPONSE: We added several sentences to list all the papers gathered during our literature review in Section 2.5 and the supplementary methods on the literature review.

I find the introduction and discussion to be too sparse on references to previous work. There are several studies available on brain-wide mapping of various cell types. The authors should discuss how their approach differ from and compares to other approaches.

OUR RESPONSE: We added additional references to previous work on whole-brain cellular composition and added sentences to explain how our approach differs from these studies (see Section 1. and 4.1).

Figure 1 shows the steps of the BBCAv2 workflow with input (Data) and output (Results) of each step in different rows. It is stated in the text that "each step of this pipeline builds on the results of the previous step", but this is not entirely clear from the figure because the wording is not consistent across panels. For example, it also seems that the three data points in step 4 correspond to the output, respectively, from steps 1-3, but the points are phrased different from the results in the individual panels.

OUR RESPONSE: The wording in the Figure 1 was indeed confusing in some boxes. We rephrased the sentences in the figure to make it clearer.

The variable use of terms like “thresholded”, “filtered”, and “binarized” images in the methods section is somewhat confusing

OUR RESPONSE: We used 3 versions of the ISH images. The raw, filtered and the binarized versions correspond respectively to the Figure 3 panels A, B and C. The “thresholded” term in the main text was incorrect and corrected it for “filtered” instead.

Reviewer #2:

1) Spell out clearly, possibly in the introduction, that the atlas is itself a model. The atlas is not just a collection of elements, but it also requires an interpolation from available data.

OUR RESPONSE: We added a few sentences in the introduction (see Section 1.) to clearly state that the result of our pipeline is a model.

2) Explain clearly what is not available from data and requires an advanced atlas reconstruction.

OUR RESPONSE: We added some explanations in the introduction (see Section 1.) to clearly state which data can and cannot be retrieved from the literature on cell composition.

3) How was the literature search carried out? It is not clear if all the paper used for model correction (e.g., table 1) are reported in the references. If not, a complete list could be added in the supplementary material

OUR RESPONSE: We added a Section in the supplementary materials to explain our methods to extract literature values to extend the excel file descriptions.

4) A glossary would be useful, since there are several non-standard abbreviations that are not always easy to track. E.g., ISH, AV, AIBS, CCF, BBCA, etc.

OUR RESPONSE: We now added a reference in Section 1.1 to the table of abbreviations in the supplementary materials.

5) Fig.5: x-axis is marker intensity? please specify.

OUR RESPONSE: The x axis of Figure 5 corresponds to the mean region intensity. We specified the x and y axis labels in the caption.

6) Fig.6: a higher resolution would be useful

OUR RESPONSE: We increased the resolution of Figure 6 to make the cell placement more visible.

7) The main result is the comparison of v1 to v2 of the BBCA. This should be discussed more thoroughly. In general, physiological, and anatomical implications are somehow diminished compared to the methodological part. A better discussion of results is needed. For example, table 4 shows interesting predictions on the inhibitory neuron populations in different brain regions. A quantitative mapping to known neuronal populations is possible

in several cases and should be discussed. E.g., for cerebellum inh/neu=13% seems unusually low but it matches the presence of a huge population of excitatory granule cells. In striatum, inh/neu=81.4% is unusually high and matches the predominance of inhibitory cells. Etc. On the same line, a homologous of Fig.8 should be done also for cerebellum and basal ganglia.

OUR RESPONSE: We thank the reviewer for this comment. However, in this manuscript, we do not aim to analyze the impact of our change of method in the 955 unique regions of the brain. Instead, we focused on regions where we observe major changes between BBCAv1 and BBCAv2 and/or found disparate results (based on Figure 6AB and Figure 10AB). We added a few explanations of the results and the changes between BBCAv1 and BBCAv2. See Section 3.4 for an explanation on the hippocampus, dentate gyrus results, Section 4.8 for explanations for the thalamus, cerebellar cortex, and striatum. In short, our density estimates for the hippocampus agree with literature and differences observed in Figure 10AB are linked to excitatory neurons of the dentate gyrus expressing GAD67 (Zeisel et al. [1]). In the cerebellar cortex and in the thalamus, Zeisel et al. reported that the inhibitory population is co-expressing GAD67, PV and SST which means that we should refine our assumptions. Finally, the striatum's neuron population is almost purely inhibitory (see review from Tepper et al. [4]).

8) Fig. 9 should be better commented. In 9c, the increase of literature data should not increase R2 ?

OUR RESPONSE: We simplified the panel C of the Figure 9 to display the average of each fitting group instead of displaying each of them and adapted the caption accordingly, this will make it easier for the reader to understand it. As discussed in the main text, no, the R2 value does not increase when more literature values are added to the fitting. This indicates that the quality of the fitting does not depend mainly on the quantity of literature values used but either from the quality of the ISH datasets, or the densities of cell and neuron obtained at step 2 of the pipeline, or the method to group the points fitted. We have addressed this in more detail in the main text.

Reviewer #3:

The latest incarnation of that atlas, "CCF version 3" is widely used in the community to register a wide variety of data to, including 10,000 fully reconstructed projection neurons, and I am a bit disappointed that the authors did not find a way to map their data onto this template, using an older version of the atlas instead.

OUR RESPONSE: Our pipeline can be used with any reference volumes pair or CCF version. In our analysis, we however show that the worst results in terms of cell density estimates is obtained with the CCFv3 because of the misalignment of the AV3 to the Nissl2 (see Section 2.2). However, since the AV3 shares many regions with the previous AV versions, the results obtained in this manuscript can be directly transplanted to the AV3.

The authors motivate their choice by pointing out that the Dong NISSL sections, needed for the cell density pipeline, have not been registered well to CCF3, especially when looking at the borders of cortical layers. I do accept this choice for the scope of the current manuscript but hope that they will follow up on the plan outlined in the discussion to re-register the NISSL sections to CCF3 in such a way that the borders of cortical layers match those of the annotation.

OUR RESPONSE: We are currently exploring methods to realign datasets from the CCFv2 to the CCFv3 in order to be able to use all annotation volumes. The major difficulty that we are currently facing is linked to the shape and position of some subregions that drastically changed between versions. We hope to register every future Blue Brain Cell atlas results to the AIBS CCFv3.

As for the cortical layer border alignment issue, have you tried to work with an exclusion zone, i.e., not take into account data that is very close to a cortical layer border?

OUR RESPONSE: We thank the reviewer for the interesting suggestion. The layer border alignment issues impact L1 the most because of the large difference in cell densities between L1 and L2. However, L1 is very thin, and an exclusion zone could reduce the annotated zone too much. We are, however, exploring other techniques to automatically redraw the annotation borders in regions where we observe large differences in densities.

Another concern that I have is that the authors combine literature values from many sources, but in absolute numbers I think one of these references stands out from the crowd with head and shoulders, namely Kim et al. 2017. I have some experience with other databases that combine literature values, and this can create problems with citation scores: if all subsequent studies would cite the current study for cell densities and not the extensive work of Kim et al. 2017, then the latter author would not get sufficient credit for his work.

This can be taken care of in a citation policy that points out the terms of use of the database and which studies must be cited depending on how the database is used. Please consider adding such a policy to the manuscript and website.

OUR RESPONSE: This is a very valid concern. We have added a paragraph in our Acknowledgements and in our literature review section (see supplementary methods) to explain to readers how to properly cite values in our review and to have a particular attention to the Kim et al. [6] paper.

Line 166: "are not sufficient to estimate the density of all inhibitory subtypes in each voxel of the mouse brain"

=> mention voxel size, 10, 25 or 100 microns?

OUR RESPONSE: The voxel size of our ISH dataset could be of any size (here we are using 25 μm^3 voxel-based datasets) but the ISH slices are cut every 200 µm which impose a low resolution of the information in one direction. We added this information to the text.

Line 169: "Since several AV and Nissl volumes were recently released [33],"

=> I don't think [33] introduced a new Nissl volume, but rather an averaged STP dataset.

OUR RESPONSE: This sentence was indeed lacking a reference to the Nissl2 volume (here a technical white paper from the Allen Institute [7]) since Wang et al. [8] describes only the CCFv3 annotation volume.

Near line 172, Figure 1, row 3: "Compute a number of neuron expressing..."

=> compute the number of neurons expressing...

and same Fig row 4:

Estimates counts

=> Estimated counts

OUR RESPONSE: We thank the reviewer for spotting these errors. These have been corrected in our updated manuscript.

Near line 232, Figure 2 part D: “Normalized Neuron Densities”

=> Normalized with respect to what, and why choose a different axis than in part C?

OUR RESPONSE: The normalization of the Figure D was indeed not properly explained in the Figure caption. We added a sentence to correct this issue. Normalization was important to show in the same figure the neuron densities of the granular layer and the molecular layer. Indeed, the granular layer densities are an order of magnitude greater than the ones from the molecular layer.

Line 267: “CCFv3 version (Nissl2 + AMBA3) from the AIBS [33].”

=> Previously you used the term AV3, now AMBA3, you probably did not apply your change throughout the document.

OUR RESPONSE: This is indeed confusing; we changed the term to be AV3.

Line 268: “For each pair of reference atlases, ...”

=> Confusing, an atlas is a combination of one or more imaging modalities (space) and one or more delineations (annotation).

I think what you mean is "For each variant of the reference atlas, ..."

OUR RESPONSE: We meant to refer to the Nissl volume and Annotation volume pairs. The term “atlas” was indeed not appropriate here. We changed it for “volume”.

Line 281: “(see S1 Fig.)”

Missing Figure number

OUR RESPONSE: Here we are referring to the first supplementary figure. According to Plos Computational Biology guidelines, the supplementary figures should be referred like this: https://journals.plos.org/ploscompbiol/s/supporting-information#loc-item-description

Line 309: “In brief, this algorithm constructs a displacement field by finding pixel by pixel correspondences between an image and a reference. Applying these displacement fields as geometric transformations, images are aligned to the same reference and then converted to a 3D volume at the same resolution using downsampling.”

=> I suggest leaving out this paragraph, since you explain the procedure again in much clearer terms in the next paragraph.

OUR RESPONSE: We took into account the reviewer’s suggestion and applied it to our text.

Line 322: “For each dataset, every coronal section is automatically realigned to the corresponding anatomical section of the Nissl-stained mouse brain using the Krepl algorithm [31].”

=> Knowing that this is not the subject of this manuscript: how does the Krepl algorithm find which is the best section in the Nissl volume that corresponds to a coronal section from an ISH dataset? Also, in a quick scan of the Krepl paper I could not find this.

Is the procedure 'just' a 2d-2d registration, assuming that the Allen metadata is correct insofar slice pairing is concerned?

OUR RESPONSE: This part of the process was indeed not clear enough. The ISH brain slices, the AV2, and the Nissl2 volume have been registered within the AIBS average brain (see Kuan et al. [9]). Thus, the ISH coronal section ids should correspond to the Nissl2’s. This means that we do not have to map the position of the ISH coronal slices for the Nissl2 before running the algorithm from Krepl et al. [5]. However, for the Nissl volume used in this manuscript, for each ISH coronal slice, we manually selected the best corresponding reference Nissl slice before realigning them. These details have been added to the manuscript Section 2.3.

Line 429: “For some regions such as the CA1 field of the hippocampus, we observe a large variability of the literature estimates (see Fig. 4B): spanning from 317 GAD67+ cells per mm computed from 431 Han et al. [54] to 7166 GAD67+ cells per mm according to Jinno et al. [55]”

=> You do list factors that could contribute to the large spread in these estimates, but it is still unsatisfactory to not know which number in this particular example is the wrong one and why. It is worthwhile to contact the authors of the conflicting numbers to clarify the cause, there can be confusion about units (in the Han et al. paper they seem to mix neurons/mm2 with 'number of neurons').

This may also help with the poor fit in Figure 5 for the GAD/Rest subplot.

OUR RESPONSE: In the Han et al. [10] and Calfa et al. [11] papers, the mean density values extracted were respectively very low and very high when compared to all other literature mean values in the same regions (at least 5 times smaller/bigger). These two papers were therefore not taken into account for the fitting since their finding was conflicting. If we can clarify this issue with the original authors, we will update our results and use these data points as well.

Han et al. [10] counted GAD67 neurons from coronal brain slices (see their Table 1.). To convert these counts into densities, we estimated the area in which the count was performed. We took the average area size of all potential coronal slices within the AV for each region of the paper and divided the counts of the paper with this value. The authors mention that they use the Paxinos and Watson [12] rat brain atlas instead of the AIBS AV to delimit the contour of the brain regions which might explain the density estimates obtained.

Calfa et al. [11] provided counts of GAD1 neurons per mm2, computed from 25 µm thick coronal slices of the hippocampus, field CA3. Dividing this number by the thickness of the slice in mm3 gives us results far greater than any other literature sources measuring the same value, even considering the dorsal, ventral and middle parts as in Jinno et al. [13].

We added this information to the main text (see Section 2.5) and our new supplementary methods section on the literature review.

Line 508: Use of the term 'orig' for your estimates: I first thought this was inspired by the 'original ISH data of the Allen Institute', but when reading on it turns out that you are going to correct this with constraints and want to distinguish 'original' from 'corrected'.

I would avoid using the word 'orig' as a symbol for your model predictions, rather use a greek letter.

In the text you could replace 'original' with unconstrained.

OUR RESPONSE: We thank the reviewer for their suggestion. We replaced the references of 'original' with ‘unconstrained’ and the ‘orig’ variable with the η letter.

Paragraph 2.6: You do not make sufficiently clear why you first "count every cell reacting to the markers." in the ISH data, then convert this to an all-or-none voxelized image, and then use the transfer functions to convert back to cell count.

Why can't the counted cells in the ISH data be used directly?

OUR RESPONSE: This sentence was badly formulated, and we thank the reviewer for pointing it out. We just wanted to specify that we are considering every cell reacting to the markers, no matter their level of ISH expression. In this manuscript, we are not using a counting algorithm to extract cell counts from ISH datasets. This solution was out of the scope for this study. Additionally, our whole-brain approach implies that we need to obtain estimates from neuron dense brain regions (e.g., cerebellum, granular layer, dentate gyrus, granular layer). However, as pointed out in Erö et al. [2], counting techniques are not always producing satisfactory results especially in these dense regions because of cell overlapping and insufficient image resolution (see Figure 1 and Figure 7 in Erö et al. [2]). We reformulated this sentence (Section 2.6)

Figure 6, end of caption: "The variation of the distribution of neurons is following the original distribution of Nissl expression. The Nissl expression drops significantly after the blue line which leads to a similar decrease of cell counts in the resulting BBCAv2."

=> This is a remark that requires much more attention.

When looking at Fig 6C there are many places where the NISSL staining is darker/lighter,

so why is the level where you show the blue line so special?

And if the NISSL data is used for counting cell bodies, why does it matter whether the staining is darker/lighter?

Are you saying here that the NISSL data is wrong and that your model is also wrong?

Probably not but that is how it reads.

OUR RESPONSE: The change observed at the blue line of Figure 6AC corresponds to a significant drop that occurs after a specific Nissl brain slice and appears in every region of the brain crossing the slice (see S4 Fig.). It is most probably an artifact in the original Nissl volume. This artifact has, however, a strong impact on our final model since the cell density is following Nissl expression (see Figure 6C). The Nissl volume as well as all the data we used to build our model comes with artifacts that affect our density results. In this manuscript, we list all these artifacts so that we can explain issues in our model and discuss potential improvements. For clarity, we removed the last part of the caption and removed the blue line from the figure. Indeed, the discussion on this artifact comes up in the discussion and S4 Fig. is showing it better.

Line 609: “The result is an updated mouse cell atlas which provides a 3D position and a type label for each cell within the CCFbbp AV (see Fig. 6CD).”

=> The result is a table with predicted densities and standard deviations for each cell class in all regions of the AV.

A realization of this model with randomly placed cells within each brain region was computed and published as the Blue Brain Mouse Cell Atlas.

Please choose wording such that it becomes clear that the BB Cell Atlas represents a single realization of your model.

OUR RESPONSE: This is a good point; we should make it clear that the Cell Atlas provides cell densities for each brain voxel and that the random positions were used for visual purposes. We added a few sentences to explain this fact (Section 3.1).

Line 614: The link https://bbpgitlab.epfl.ch/nse/atlas-building-tools points to an inaccessible location: "Unable to connect"

OUR RESPONSE: The code linked to the pipeline was in the process to be published by our lab. We also switched to github. Here is the new link: https://github.com/BlueBrain/atlas-densities. The link was updated in Section 3.1 and in the conclusion.

Line 652 and 654: "We predict that 25% of the neurons of the mouse's isocortex to be inhibitory"

=> grammar error, predict that ... is or predict ... to be …

OUR RESPONSE: We thank the reviewer for noticing this error. We corrected it (Section 3.3).

Line 679-683: It is not immediately clear *why* you studied the density profiles wrt. pia so extensively, resulting in Fig. 8CD and an extended methods section. I think it is better to start by saying that Meyer et al. studied this profile and that you wanted to see whether the model is consistent with that.

OUR RESPONSE: We agree with the reviewer and reformulated our paragraph in Section 3.3.

Line 748: "Additionally, the granule layer of the dentate gyrus is clearly visible on the original experiment but not in the BBCAv2 brain slice (center black arrow)."

=> This prominent difference is not explained or discussed in the text. This layer is distinct part of the Annotation Volume, so how can it be that the model does not predict a correct value for it?

OUR RESPONSE: The inhibitory neuron density estimates of our cell atlas for the dentate gyrus granule layer are in the same range as the literature. The differences observed in Fig. 10AB comes from the cells reacting to the GAD67 marker. Indeed, Zeisel et al. [1] found that the excitatory neuron population in this region was also expressing GAD67. This region being very dense in terms of neuron densities (Erö et al. [2]), the excitatory population takes an important part in the overall GAD67 expression of the region. This is confirmed by a manual inspection of GAD67 and GAD65 sagittal slices, since the excitatory population does not express GAD65.This region is therefore an exception to our assumptions and should therefore be treated separately. We added a paragraph in Section 3.4 to explain this issue and a supplementary figure to show the GAD65 and GAD67 expression in the region.

Line 780: "It is therefore usually underestimating the densities of each neuron type according to literature, since the neuron and inhibitory neuron densities act as maximum constraints."

From Fig. 10E, I don't see that the new model underestimates densities.

And, in the cortex the inhibitory neuron density is 25% according to the model vs. 20% in Literature. That is an overestimation, not underestimation.

Be more specific with the statements so that this confusion is avoided.

OUR RESPONSE: This sentence was indeed not correct. We replaced it with “It is therefore usually providing density estimates closer to their literature counterparts for each neuron type” (Section 3.4).

Line 851: “The marker datasets, despite having been processed by the AIBS, are still subject to artefacts”

and noise which will artificially raise our cell counts estimates (see Fig. 3A).

=> I see artefacs in 3A, but not in 3C. It could also lower the estimates, isn't it?

OUR RESPONSE: Figure 3A represents the raw images from which Figure 3B (filtered image) and 3C (binarized image) were derived. The filtered image removes the background which partially corrects some of the artifacts that we see in Figure 3A. Then we realign and threshold the filtered image to obtain 3C. This step can also limit the impact of the remaining artifacts from 3B, but any major artifact such as bubbles or tearing will still be visible after all this process. True, artifacts such as holes can lower locally the expression of a marker. We now mention this in the manuscript (Section 4.3).

Line 852: “To counter these effects, Erö et al. [14] used the Nrn1 marker, as it is expressed in excitatory neurons, to limit the expression of GAD67.”

=> How can the expression in one experiment be limited by the expression of another substance in another animal? This needs more clarification. Is it because you assume that the GAD67 expression density must not be higher than some preset fraction of the Nrn1 expression density?

OUR RESPONSE: This part indeed needs clarifications. In Erö et al. [2], the authors normalized the expression of GAD67 with the sum of the expression of GAD67 and Nrn1 and multiplied the result with the ratio of inhibitory neurons using global counts from Kim et al. [6]. The resulting dataset is therefore limited by the expression of Nrn1 and the expected ratio of inhibitory neurons. We added these specifications to our manuscript (Section 4.3).

References:

1. Zeisel A, Hochgerner H, Lönnerberg P, Johnsson A, Memic F, van der Zwan J, et al. Molecular Architecture of the Mouse Nervous System. Cell. 2018;174: 999-1014.e22. doi:10.1016/j.cell.2018.06.021

2. Erö C, Gewaltig M-O, Keller D, Markram H. A Cell Atlas for the Mouse Brain. Front Neuroinformatics. 2018;12. doi:10.3389/fninf.2018.00084

3. Lee S, Hjerling-Leffler J, Zagha E, Fishell G, Rudy B. The Largest Group of Superficial Neocortical GABAergic Interneurons Expresses Ionotropic Serotonin Receptors. J Neurosci. 2010;30: 16796–16808. doi:10.1523/JNEUROSCI.1869-10.2010

4. Tepper J, Tecuapetla F, Koos T, Ibanez-Sandoval O. Heterogeneity and Diversity of Striatal GABAergic Interneurons. Front Neuroanat. 2010;4. Available: https://www.frontiersin.org/article/10.3389/fnana.2010.00150

5. Krepl J, Casalegno F, Delattre E, Erö C, Lu H, Keller D, et al. Supervised Learning With Perceptual Similarity for Multimodal Gene Expression Registration of a Mouse Brain Atlas. Front Neuroinformatics. 2021;15: 691918. doi:10.3389/fninf.2021.691918

6. Kim Y, Yang GR, Pradhan K, Venkataraju KU, Bota M, García del Molino LC, et al. Brain-wide Maps Reveal Stereotyped Cell-Type-Based Cortical Architecture and Subcortical Sexual Dimorphism. Cell. 2017;171: 456-469.e22. doi:10.1016/j.cell.2017.09.020

7. Allen Institute for Brain Science. ALLEN Mouse Brain Atlas. Technical white paper: Informatics data processing. In: Allen Institute for Brain Science [Internet]. 2011. Available: http://help.brain-map.org/download/attachments/2818169/InformaticsDataProcessing.pdf

8. Wang Q, Ding S-L, Li Y, Royall J, Feng D, Lesnar P, et al. The Allen Mouse Brain Common Coordinate Framework: A 3D Reference Atlas. Cell. 2020;181: 936-953.e20. doi:10.1016/j.cell.2020.04.007

9. Kuan L, Li Y, Lau C, Feng D, Bernard A, Sunkin SM, et al. Neuroinformatics of the Allen Mouse Brain Connectivity Atlas. Methods. 2015;73: 4–17. doi:10.1016/j.ymeth.2014.12.013

10. Han L-C, Zhang H, Wang W, Wei Y-Y, Sun X-X, Yanagawa Y, et al. The Effect of Sevoflurane Inhalation on Gabaergic Neurons Activation: Observation on the GAD67-GFP Knock-In Mouse. Anat Rec Adv Integr Anat Evol Biol. 2010;293: 2114–2122. doi:10.1002/ar.21113

11. Calfa G, Li W, Rutherford JM, Pozzo-Miller L. Excitation/Inhibition Imbalance and Impaired Synaptic Inhibition in Hippocampal Area CA3 of Mecp2 Knockout Mice. Hippocampus. 2015;25: 159–168. doi:10.1002/hipo.22360

12. Paxinos G, Watson C. The Rat Brain in Stereotaxic Coordinates. Elsevier; 1982. doi:10.1016/C2009-0-63235-9

13. Jinno S, Aika Y, Fukuda T, Kosaka T. Quantitative analysis of GABAergic neurons in the mouse hippocampus, with optical disector using confocal laser scanning microscope. Brain Res. 1998;814: 55–70. doi:10.1016/S0006-8993(98)01075-0

All the authors have read and approved the manuscript's modifications according to Editors and Referees criticisms and suggestions. We would like to confirm that this full paper has not been submitted to another journal for publication and has not been previously published.

All the authors have read and approved the final manuscript. The authors declare that there are no competing interests and that they have received no funding for this study. We are grateful to the Reviewers for their careful reading and hope that the manuscript now fulfills the criteria for publications in *Plos Computational Biology*.

Sincerely yours,

Corresponding author

Dimitri Rodarie, PhD Student
Blue Brain Project / EPFL
Campus Biotech
Chemin des Mines 9
CH-1202 Geneva
Email: dimitri.rodarie@epfl.ch
Tel: +41 21 69 55 180
