# Supplementary material for: A method to estimate the cellular composition of the mouse brain from heterogeneous datasets
Source: PLoS Comput Biol. 2022 Dec 21;18(12):e1010739. doi: 10.1371/journal.pcbi.1010739 (PMC9838873; doi:10.1371/journal.pcbi.1010739)
Supplement: Attachment — Submitted filename: Response_to_reviewers.docx [file pcbi.1010739.s014.docx]

October 6^th^, 2022

Subject: Revision and resubmission of manuscript PCOMPBIOL-D-22-00098

Dear Editor in chief,

Please find attached a re-submission of the revised manuscript entitled: “A method to estimate the cellular composition of the mouse brain from heterogeneous datasets”, as a full paper. We wish to thank all the Reviewers for their constructive and very helpful suggestions. We kindly provide our responses to the Reviewers’ comments and believe that our revised manuscript has been improved and may now be suitable for publication in *Plos Computational Biology*. The modifications have been highlighted in green in the final manuscript.

# Reviewers’ comments:

## Reviewer #1:

**Overall, I think the authors in this revision have provided important clarifications for readers to understand their approach, as well as interesting new observations to support their data. I do, however, have some remaining comments that should be addressed:**

**- I think the revision help clarify many of the limitations and advantages of the authors approach related to the transfer function and density estimates, and understand the authors’ point that the literature variability presents a difficulty for the fit better after this revision. However, the authors have not really addressed my concern that the density estimates may be biased by the method used to compute them. This is in a way parallel to how estimates in the literature vary a lot, probably because such different methods are used across studies. Thus, I still believe the authors should discuss how their GAD to subtypes ratios could be affected by the fact that brain-wide data from a single study were available for all three subtypes but not the GAD cells.**

OUR RESPONSE:

We understand that the reviewer is concerned by the impact the data source has on our density estimates. The reviewer rightfully pointed out that the estimates for the estimates of the three inhibitory subtypes PV+, SST+ and VIP+ are mostly derived from literature and in particular the Kim et al. study [1] (see Section 2.5). This study alone provides a great proportion of the literature values used as constraints by the pipeline. Moreover, the PV+, SST+ and VIP+ density estimates that we extracted from this study cover the entire brain and are consistent with each other (same methodology to count cells).

For GAD67, however, no such data were available, and we had to combine data from different studies. Our resulting GAD67+ estimates are noisier than those of PV+, SST+ and VIP+ because of the greater variety of paper and method used to obtain the estimates but also because of the variability induced by the fitting (see Sections 4.5 and 4.6).

All first density estimates are then optimized all at once to fit in the same framework or cell atlas model (see Section 2.7). The variability of each first estimate is however acknowledged since we collected a standard deviation value for each value. Hence, a weight is assigned to each first estimate based on its reliability (see Section 4.7.1); here we chose the ratio of the mean value over its standard deviation. This solution gives more credit to reliable values. This way, we also minimize the bias towards any literature or fitting source, or any method to extract the first estimates in general with our optimization algorithm.

As a result, our final PV+, SST+ and VIP+ neuron density estimates are very close to the ones from Kim et al. because of their amount, coherence, and reliability. Any bias present in the Kim et al. study would impact largely our estimates for the three inhibitory subtypes. This bias can be resolved by the integration of more literature values on PV+, SST+ and VIP+ neurons from different studies.

Additionally, our method to estimate inhibitory neurons introduces a bias for the Rest population. By definition, the estimates of the Rest population are defined according to the proportion of inhibitory neurons that is neither PV+, SST+ nor VIP+. This particular population can be related to the GAD67 to subtypes ratio the reviewer is referring to (ratio = PV+SST+VIP+REST / PV+SST+VIP). As we discussed in Section 4.7.3, our counts of PV, SST and VIP are reliable because of the quality of the data used to constrain them. This means that the reliability of our Rest estimates depends mostly on the GAD67+ densities and should be considered with the same carefulness.

We thank the reviewer for bringing our attention to this topic which was indeed not discussed enough in our manuscript. We provided more details on how each first estimate is weighted in Section 4.7.1. We also have written a new paragraph in Section 4.7.3 on our bias due to the Kim et al. study. Additionally, we added precisions on the method bias for the Rest population in Section 4.8.2. Finally, we created headings in most of our Discussion Sections to guide the reader towards the particular points discussed.

.

**- I appreciate the added observations in section 3.4. and the new S7 Fig, and think it adds balance to the qualitative comparison. However, Figure S7 does not say which image represents which staining. It is also not clear to me what the authors are referring to by the "other population of cells", smaller in diameter and lower GAD67 population. It would be useful if they could add labels and pointers in the figure to more clearly indicate their observations. Lastly it should be made clear which Allen brain atlas experiments and slices are depicted in this figure, and they should be cited in the main text with links so that the reader can easily go in and look at the high-resolution images.**

**- Similarly, the sagittal section from the AIBS shown in figure 10 should also be cited appropriately with experiment ID so readers can find it.**

OUR RESPONSE: We thank the reviewer for bringing our attention to the lack of precision in our Figure captions. We have added to our Figure captions, the experiment ids and the slice number of each ISH experiment used (see Fig. 3, Fig, 10, S7 Fig., S8 Fig., and the Acknowledgements Section). We also added some arrows to point to the different cell types discussed in S7 Fig, as suggested by the reviewer.

**- I think the language should be critically reviewed across the manuscript, and especially within the newly added text, where there are quite a number of typos and poorly formulated sentences.**

OUR RESPONSE: We thank the reviewer for the comment. We have reviewed the sentences of our manuscript and made the required corrections.

# References:

1.	Kim Y, Yang GR, Pradhan K, Venkataraju KU, Bota M, García del Molino LC, et al. Brain-wide Maps Reveal Stereotyped Cell-Type-Based Cortical Architecture and Subcortical Sexual Dimorphism. Cell. 2017;171: 456-469.e22. doi:10.1016/j.cell.2017.09.020

All the authors have read and approved the manuscript’s modifications according to
Editors and Referees criticisms and suggestions. We would like to confirm that this full
paper has not been submitted to another journal for publication and has not been
previously published.

All the authors have read and approved the final manuscript. The authors declare that
there are no competing interests and that they have received no funding for this study.
We are grateful to the Reviewers for their careful reading and hope that the manuscript
now fulfills the criteria for publications in *Plos Computational Biology*.

Sincerely yours,

**Corresponding author**

Dimitri Rodarie, PhD

Blue Brain Project / EPFL

Campus Biotech

Chemin des Mines 9

CH-1202 Geneva

Email: [dimitri.rodarie@epfl.ch](mailto:dimitri.rodarie@epfl.ch)

Tel: +33 63 63 56 254
